# Synthesis of enantiomerically pure alcohols and amines *via* biocatalytic deracemisation methods

**DOI:** 10.1039/C9CY01539F

**Published:** 2019-09-03

**Authors:** Musa M. Musa, Frank Hollmann, Francesco G. Mutti

**Affiliations:** a Department of Chemistry, King Fahd University of Petroleum and Minerals, Dhahran 31261, Kingdom of Saudi Arabia; b Department of Biotechnology, Delft University of Technology, van der Maasweg 9, 2629HZDelft, The Netherlands; c Van’t HoffInstitute for Molecular Sciences, HIMS-Biocat, University of Amsterdam, Science Park 904, 1098 XH, Amsterdam, The Netherlands

## Abstract

Deracemisation *via* chemo-enzymatic or multi-enzymatic approaches is the optimum substitute for kinetic resolution, which suffers from the limitation of a theoretical maximum 50% yield albeit high enantiomeric excess is attainable. This review covers the recent progress in various deracemisation approaches applied to the synthesis of enantiomerically pure alcohols and amines, such as (1) dynamic kinetic resolution, (2) cyclic deracemisation, (3) linear deracemisation (including stereoinversion) and (4) enantioconvergent methods.

## Introduction

Chiral alcohols^1,2^ and amines^3–7^ are pivotal building blocks for the manufacturing of chemical products such as agrochemicals, flavours, fragrances and pharmaceutical. This predominance of chiral alcohol and amine moieties has stimulated the development of a multitude of synthetic and physicochemical approaches including chromatographic separation of racemates on high-performance porous chiral stationary phases,^8^ enantioselective resolution using resolving agents^9^ and stereoselective synthesis from ketones and imines.^10–15^

**Scheme 1:**
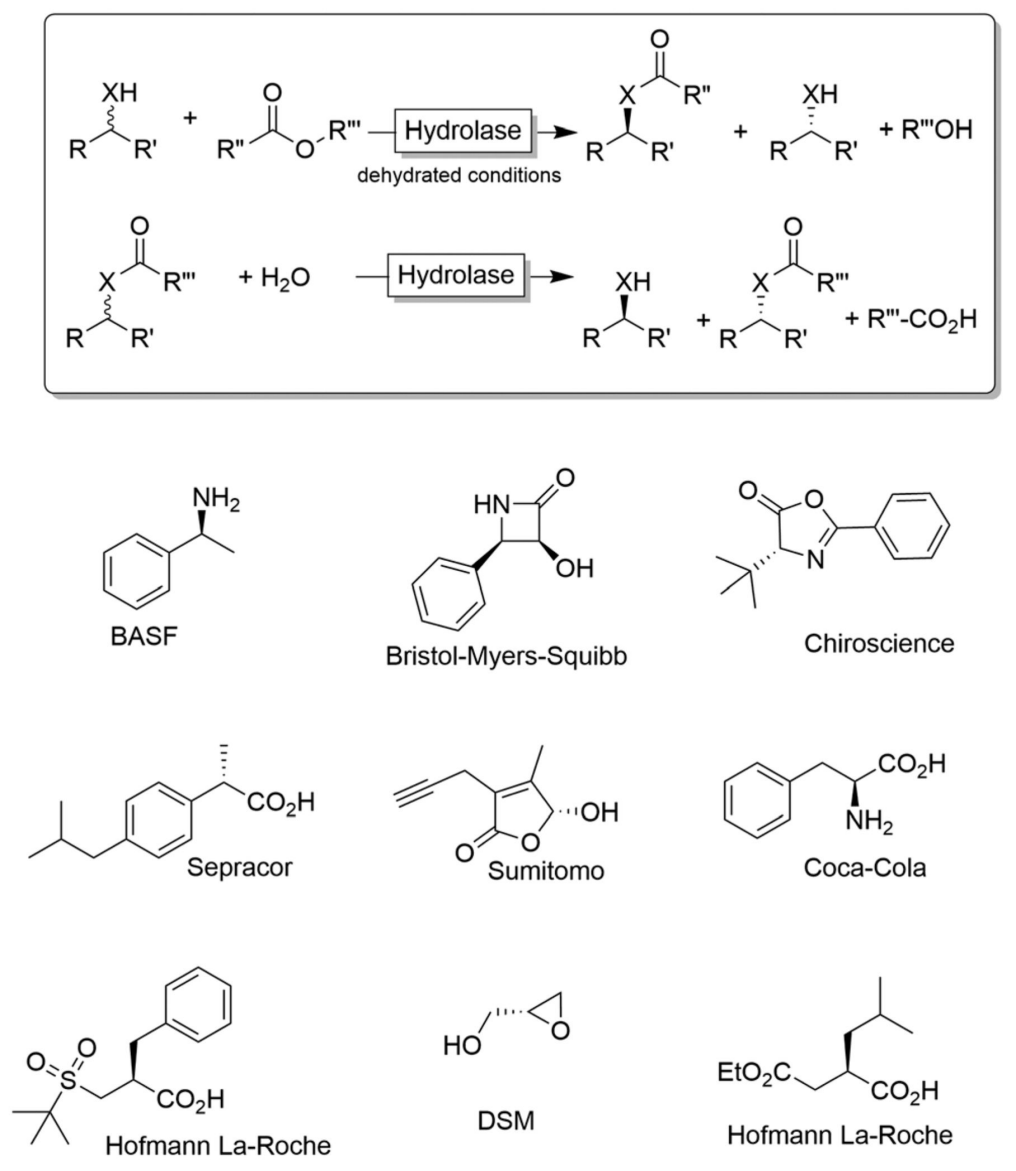
Kinetic resolution of alcohols (X = O) or amines (X = NH) using hydrolases. Under non-aqueous conditions, the synthetic direction (esterification, amidation) is preferred while under aqueous conditions the hydrolytic direction is preferred. Below a selection of industrial products obtained *via* kinetic resolution (KR) or dynamic (in this case, spontaneous) kinetic resolution (DKR) is shown.^19,20^

Due to their inherent homochirality, enzymes play an important role as catalysts in the synthesis of optically active alcohols and amines.^16,17^ Historically, hydrolase-catalysed kinetic resolution (KR, Scheme 1) reactions were the first ones to be explored.^18^ Despite the recently reduced interest in the academic world on these enzymes (especially because of the theoretical maximal yield of 50%), hydrolase-catalysed KR reactions are still largely applied on industrial scale production of such optically active compounds (Scheme 1).^19,20^

In an ideal KR reaction, only one enantiomer of a racemic substrate mixture is converted by an enantioselective catalyst. That results in the complete conversion one enantiomer into a product that can be separated physicochemically from the starting material. Principally, this transformation can occur either through retention of the chiral information *(e.g., via* (de)acylation of the heteroatom), or by destroying it *(e.g., via* oxidation of the α-chiral alcohol or amine moiety to a prochiral carbonyl or imine, respectively). In the former case, at least theoretically, the other enantiomer (product) can also be recovered, whereas the achiral product is generally discarded in the latter case. Hence, the maximal yield of 50% makes KR reactions unattractive from an environmental point of view as a significant amount of starting material per definition is waste.

In recent years, enantioselective reactions yielding enantiomerically pure alcohols and amines from their prochiral carbonyl and imine starting materials, respectively, have gained significant attention. Particularly, alcohol dehydrogenases (for the synthesis of optically active alcohols) as well as transaminases and imine reductases (for the synthesis of optically active amines) are well-established today. Recent advances in this area have been deliberated in some excellent recent review articles and therefore will not be further discussed here.^7,15,21–28^

**Scheme 2:**
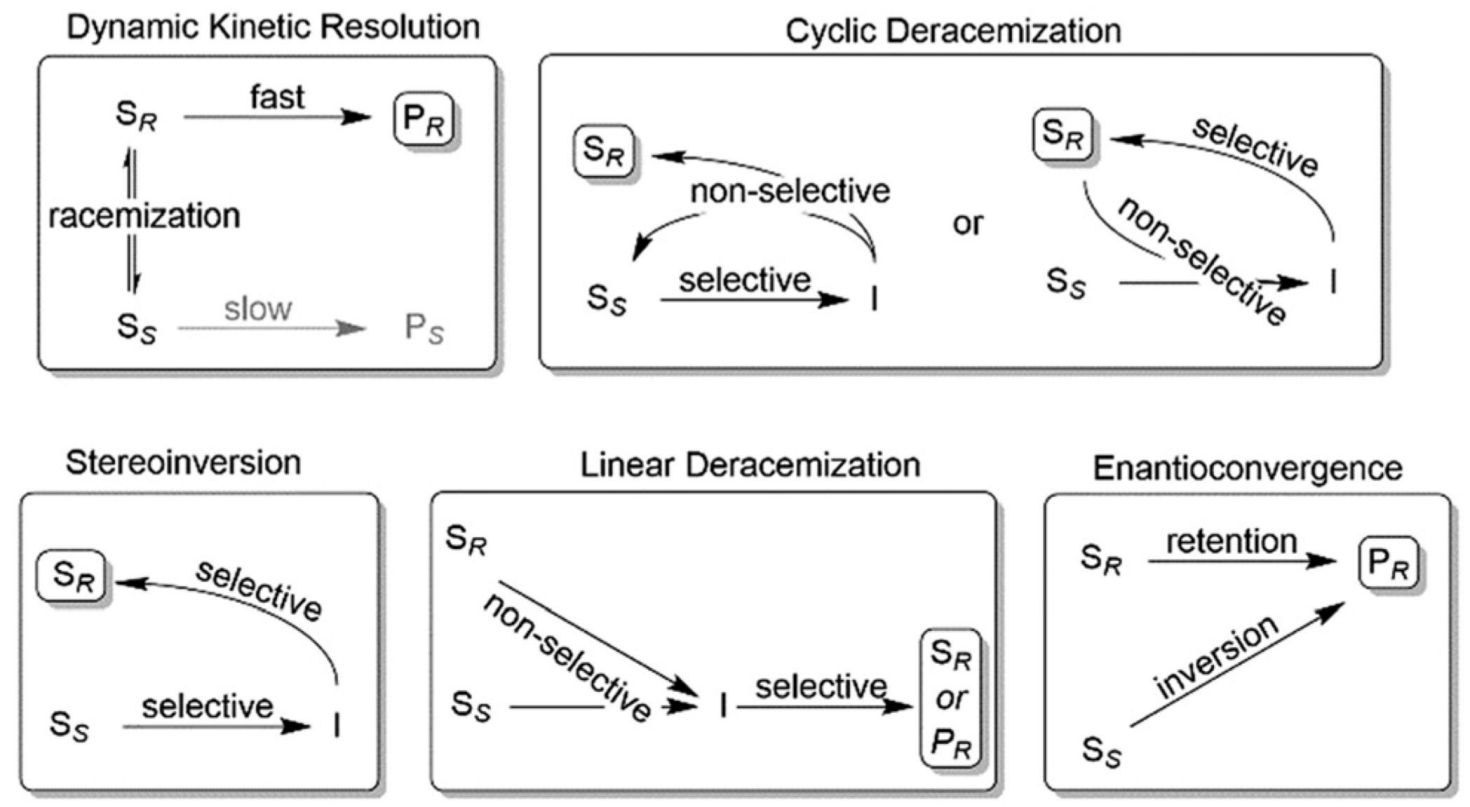
Common deracemisation methods. S: chiral starting material such as an amine or alcohol, P: product, I: prochiral intermediate such as a ketone or an imine.

Nevertheless, often, racemic alcohols and amines are more readily available than their corresponding carbonyl and imine compounds, respectively; therefore, starting from racemic alcohols or amines often represents the more straightforward and/or more cost-efficient approach as compared to the stereoselective reductions/reductive aminations mentioned above. To overcome the intrinsic disadvantage of KR reactions, a range of deracemisation protocols have been developed in the past years, which can be subdivided into different categories such as (1) dynamic kinetic resolution, (2) cyclic deracemisation, (3) stereoinversion and (4) enantioconvergent methods (Scheme 2).^29–36^

In the following sections, recent progress in each deracemisation approach is outlined and critically discussed.

## Dynamic kinetic resolution (DKR) reactions

**Scheme 3:**
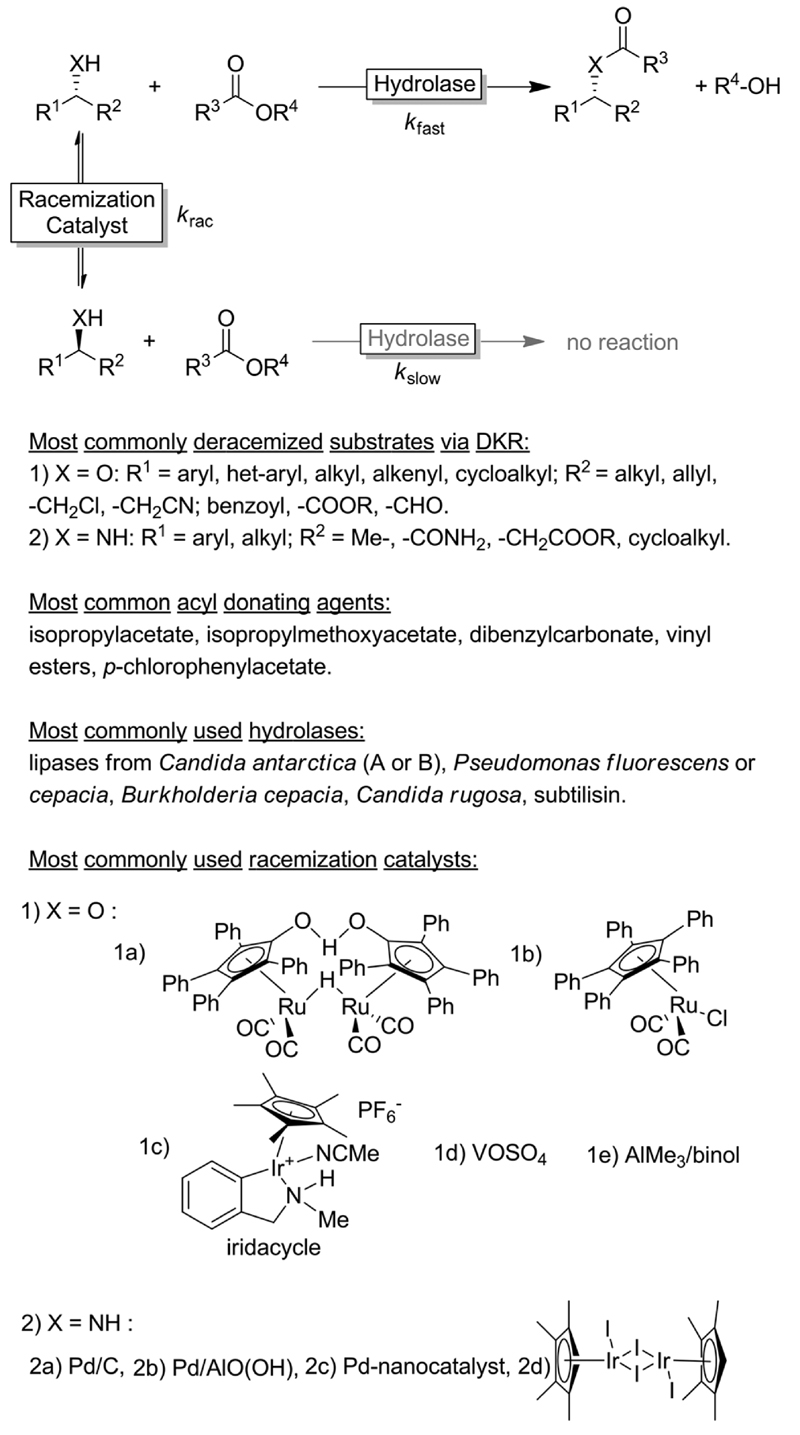
Generalised scheme of a dynamic kinetic resolution reaction involving hydrolases as KR-catalyst under non-aqueous conditions.

Dynamic kinetic resolution (DKR) protocols couple an *in situ* racemisation of the starting alcohol or amine substrate with an enantioselective (enzymatic) follow-up reaction, thus yielding a non-racemisable product (ideally in optically pure form, Scheme 3).

The selection criteria for a successful DKR were formulated by Verho and Bäckvall:^37^ (1) the KR reaction must be sufficiently enantioselective (*i.e., k*_fast_/*k*_slow_ ≥ 20); (2) both racemisation catalyst and hydrolase must be compatible (i.e., exhibiting comparable operational windows in terms of solvent, temperature *etc*. and do not mutually inactivate each other); (3) the rate of the racemisation step must be at least 10-fold higher than the rate of the enzymatic acylation step for the slow reacting enantiomer (*i.e., k*_rac_ /*k*_slow_ > 10); and (4) the racemisation catalyst must not react with the final KR product.

Since the pioneering work by the groups of Williams^38^ and Bäckvall^39,40^ on the DKR of racemic alcohols, the field has grown significantly during the past two decades, reaching a high level of maturity. Some excellent recent reviews covered the blooming field of DKR.^37,41–43^ The DKR of racemic alcohols is classically performed using transfer hydrogenation-type ruthenium catalysts.^37^ The most famous racemisation catalyst is the dimeric Ru-Shvo catalysts, whose utilisation requires relatively high temperatures and the requisite of activated aryl esters as acyl donating agents in the enzymatic step. In fact, the need for high temperature in the racemisation step restricts the possible DKR reactions to a combination of Ru-Shvo catalyst with only highly thermostable hydrolases (mainly (*R*)-selective).^44^ On the other hand, the advantage of the classical Ru-Shvo catalyst is its versatility as a wide range of structurally diverse functionalised alcohols could be deracemised through the combination with hydrolases possessing different substrate scope.^37,41,42^ However, most of the research in this field has been devoted to the development of room temperature-active racemisation catalysts.^45,46^ Although a “universally” applicable racemisation catalyst remains elusive, improved generations of transfer-hydrogenation Ru-catalysts are available^37^ along with Ir-based catalysts, the latter being particularly suitable for the DKR of halohydrins.^47^ As alternatives to transfer hydrogenation-type racemisation catalysts, other catalysts operating *via* dehydrative mechanisms such as AlMe_3_, VOSO_4_ have been implemented in DKR.^37^ These catalysts are not only cheaper than Ru-, Ir- or Pd-catalysts but also enable the DKR of tertiary alcohols, which is mechanistically impossible *via* hydrogen transfer catalysis. However, only starting materials that form stable carbocations are converted.

The DKR of amines is a significantly more challenging process because the substrate racemization catalysed by Ru-based catalysts requires temperatures above 100 °C.^37,41–43^ Under these conditions, the amine substrate can act as a coordinating ligand to the metal centre of the racemization catalyst, thus reducing the rate or even interrupting the DKR process. Furthermore, the *in situ* formed imine intermediate can react with the starting amine yielding various by-products through aminal intermediates. As these types of side-reactions are favoured only above a certain temperature, most of the research was and still is focused on developing racemisation catalysts active at temperatures of 50 °C or below. A number of catalytic systems based on Pd/BaSO_4_, Pd/CaCO_3_, Pd/AlO(OH), Pd nanoparticles, Ir, *etc.* proved to be promising.^43^

Incompatibility of enzymes and transition metals is well noted; accordingly, the development of transition metal-independent *in situ* racemisation methods is a current focus of research. Racemases, for example, are attractive catalysts for the racemisation step in DKR due to the expected intrinsic compatibility with the enzymatic KR-catalysts. In an early contribution, Faber and coworkers reported a sequential deracemisation of mandelic acid through the combination of a lipase-catalysed KR followed by a racemase step to convert the enantiomerically pure starting alcohol back into the race-mate.^48^ Both steps had to be performed separately because the lipase-catalysed acylation was efficient in a non-aqueous medium only, whereas the racemase-step could only be performed in an aqueous medium. Nevertheless, over four consecutive cycles of KR and racemisation an overall yield of 80% of enantiomerically pure (S)-O-acetyl mandelic acid was obtained.

**Scheme 4:**
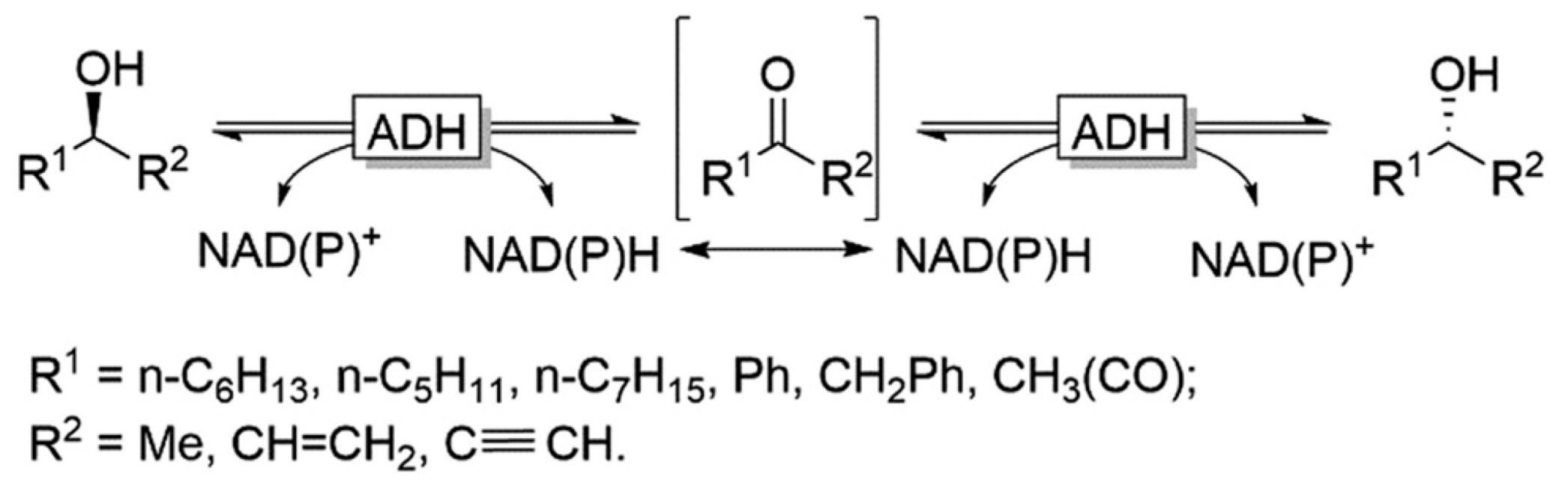
Reversible oxidation/reduction equilibrium mediated by ADHs. In the absence of a cofactor regeneration system the entropy favoured racemisation takes place. NAD(P)H: nicotinamide-adenine di-nucleotide (phosphate) reduced, NAD(P)^+^: nicotinamide-adenine dinu-cleotide (phosphate) oxidised.

Naturally, racemases that act on alcohols and amines are rather scarce, which becomes obvious considering the homochirality of *e.g.* amino acids and carbohydrates throughout all kingdoms of life. An alternative approach for the *in situ* racemisation explored in recent years is to exploit enzymes with non-exclusive enantioselectivity in reversible transformations. Alcohol dehydrogenases (ADHs), for example, have been demonstrated as efficient racemisation catalysts. In the absence of a cofactor regeneration system, ADHs catalyse the reversible equilibrium between alcohols and the corresponding prochiral carbonyl compounds. Provided that the enantioselectivity of a given ADH is not exclusive, the thermodynamically favoured (entropy-driven) racemisation will take place (Scheme 4).

This approach was pioneered by Kroutil and coworkers, who demonstrated that even highly enantiospecific ADHs such as the ADH from *Lactobacillus kefir (Lk*ADH) or *Rhodococcus ruber* (ADH-A) are not perfectly enantiospecific and will, in the absence of a cofactor regeneration system, mediate the racemisation of a broad range of enantiomerically pure alcohols.^49^ On the other hand, the use of highly enantio-selective ADHs is disfavoured from a kinetic perspective as, for instance, the racemisation of *ca*. 20 mM of (*R*)- or (*S*)-configured 2-octanol (one of the optimal substrates) requires about 30 h using elevated concentrations of the ADH.

**Scheme 5:**
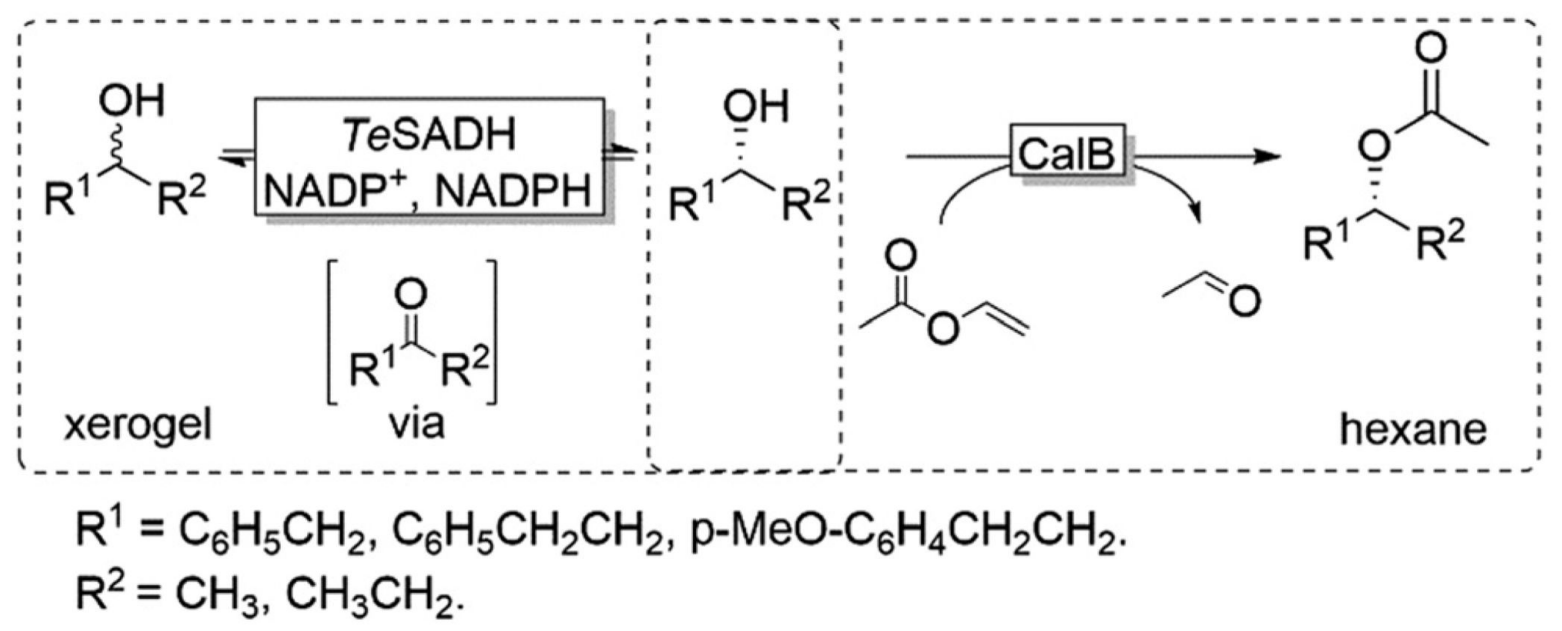
Bi-enzymatic DKR of racemic alcohols combining W110A *Te*SADH-catalysed racemisation with CalB-catalysed KR. *Te*SADH: *Thermoanaerobacter ethanolicus* secondary ADH (EC 1.1.1.2), CaLB: *Candida antarctica* lipase B (EC 3.1.1.3).

Coupling the above-described racemisation reaction to a hydrolase-catalysed KR, however, is not straightforward in aqueous media due to the favoured hydrolysis of the desired enantiomerically pure ester product. To circumvent this limitation, Musa and coworkers established a procedure utilizing xerogel-immobilised secondary ADH from *Thermoanaerobacter ethanolicus* (TeSADH, EC 1.1.1.2, more specifically its W110A variant)^50,51^ for the *in situ* racemisation of various alcohols under water-deficient reaction conditions.^52^ Under such semi-arid conditions, the lipase-catalysed KR reaction occurred efficiently, thus obtaining a bi-enzymatic DKR (Scheme 5).

Using a teabag-variation of the above-described bi-enzymatic DKR system (Scheme 5),^53^ Kroutil and coworkers also developed this approach by implementing a variant of *Te*SADH (i.e., containing the following mutations: W110A I86A C295A).^50,53^ These two reports provided a proof-of-concept demonstration for a bi-enzymatic DKR of alcohols. However, the productivity of this DKR was moderate because of diffusion limitation of the alcohol substrates, which was probably due to the complexity of the system requiring two liquid phases (organic and aqueous buffer) as well as tea-bag compartmentalisation of the lipase.

Overall, DKR reactions still represent a flourishing area of research. Currently, the transition-metal-catalysed racemisation approaches prevail but some purely enzymatic approaches are reaching maturity and preparative applicability.

## Cyclic deracemisation approaches

**Scheme 6:**
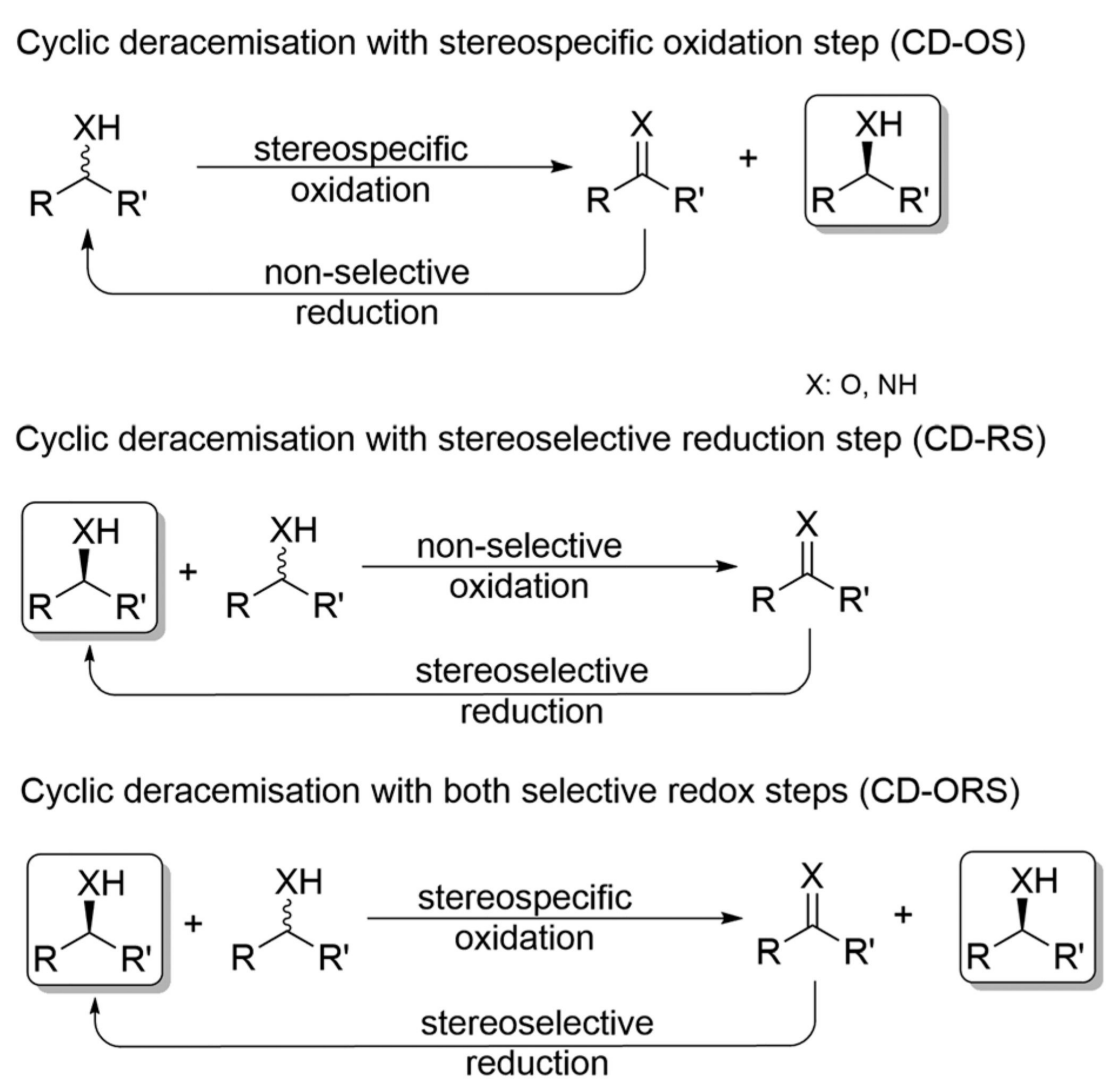
Concepts for cyclic deracemisation methods. If CD-ORS comprise a fully stereospecific oxidation and a fully stereoselective reduction, a single cycle is required thus representing the case of deracemisation *via* stereoinversion (*vide infra*).

In general, cyclic deracemisation (CD) approaches combine two redox steps in one-pot (Scheme 6).

In the majority of cases reported, a highly enantioselective or enantiospecific reaction step (commonly catalysed by an enzyme) is combined with a concurrent non-stereoselective step (often mediated by a chemical reagent). However, both oxidation and reduction steps can be, at least partly, stereo-selective. Notably, if both steps are completely stereoselective, a single cycle can lead already to full deracemisation. This particular case will be treated separately in the section deracemisation *via* stereoinversion, whereas in this section the focus will be on truly “cyclic” processes. The most common type of cyclic deracemisation reactions comprises a stereospecific oxidation step (essentially representing a KR)—thus leaving the desired alcohol or amine enantiomer untouched—while transforming the other enantiomer into the prochiral intermediate (ketone or imine). The intermediate is then chemically reduced back into the racemic starting material by a non-stereoselective reagent or catalyst. Iterations of this reaction sequence will eventually yield the enantiomerically pure starting material. If the oxidation biocatalyst is fairly stereospecific, this “cyclic deracemisation *via* stereospecific oxidative step” (CD-OS) becomes a rather efficient deracemisation method. Indeed, if the enzymatic oxidation step is enantiospecific, already after eight theoretical rounds the enantiomeric access of the starting material will exceed 99.5%.

### Cyclic deracemisation reactions with a stereoselective reduction step (CD-RS)

**Scheme 7:**
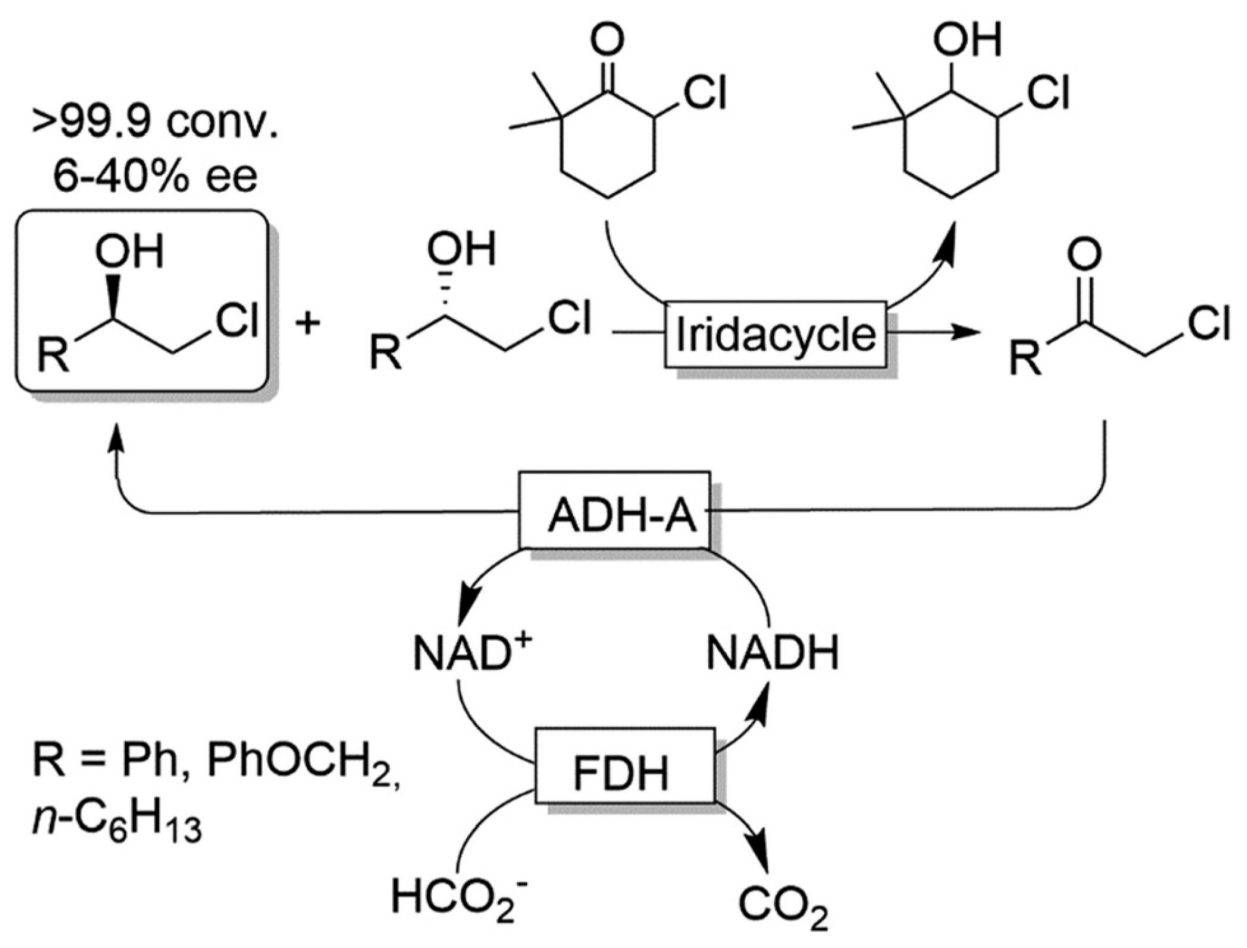
Enantioselective reductive cyclic deracemisation (CD-RS) of chlorohydrins using a concurrent iridium-catalysed non-stereoselective oxidation and ADH-A-catalysed stereoselective reduction. Structure of iridacycle is shown in Scheme 3.

Cyclic deracemisation reactions can comprise a stereoselective reduction reaction along with a non-selective oxidation step *(i.e.,* CD-RS). One obvious disadvantage of this CD approach is that, during the non-selective oxidation step, the desired enantiomer is also converted thereby lowering the overall efficiency of this approach by futile redox cycles. A proof of principle of CD-RS was provided for the partial deracemisation of chlorohydrins by combining an Ir-organocatalyst with an ADH (Scheme 7),^54^ the former of which was previously implemented in DKR for the racemisation step of chlorohydrins (Scheme 3).^47^ In this deracemisation strategy —as no ADH was known to be able to oxidize chlorohydrins to α-chloroketone intermediates—the authors envisaged to exploit an ‘outer-sphere’ oxidation mechanism catalysed by the iridacycle catalyst at the expense of a sacrificial oxidant (2-10 equiv.). Although ‘outer-sphere’ oxidation occurred, the iridacycle catalyst still performed its ‘inner-sphere’ racemisation thus partly eroding the enantiomeric excess obtained after each run of the chemo-enzymatic cascade. Therefore, the one-pot, concurrent and orthogonal cascade yielded only modest results in terms of optical purity of the final chlorohydrin. Theoretically, the system could lead to enantiomerically pure chlorohydrins by applying a redox metal-catalyst that operates solely through an ‘outer-sphere’ mechanism and by fixing the number of deracemisation cycles through the equivalents of the sacrificial oxidant.

### Cyclic deracemisation reactions with a stereospecific oxidation step (CD-OS)

#### CD-OS to produce chiral alcohols

**Scheme 8:**
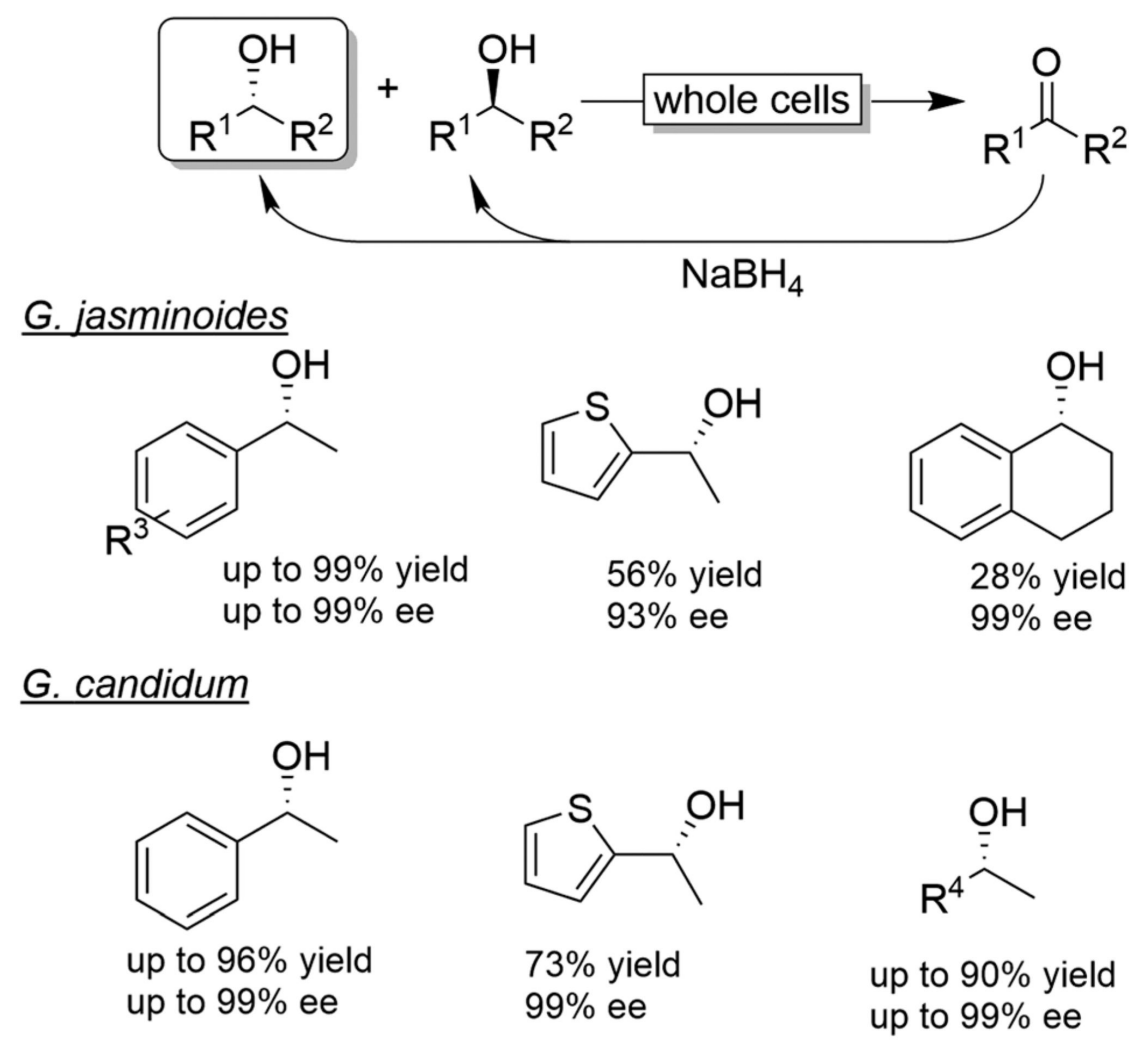
Examples for CD-OS of alcohols using *Gardenia jasminoides* or *Geotricium candidum* as KR catalysts coupled to non-selective reduction of the intermediate ketone by NaBH_4_.

A few alcohol CD-OS reactions have been reported by combining fungal or plant cells with NaBH_4_ (Scheme 8).

The chemo-enzymatic deracemisation using *Gardenia jasminoides* immobilised cells in calcium alginate with NaBH_4_ enabled the production of the (*R*)-configured alcohols in enantioenriched form.^55^ For instance, a hundred of milligrams scale CD-OS reaction starting from racemic 1-phenylethanol yielded the (*R*)-configured enantiomer in 84% isolated yield and 97% ee. However, the process of enantioenrichment required three cycles for a total of seven days. In another study, *Geotrichum candidum* cells were applied in combination with NaBH_4_ as a reducing agent in ionic liquids.^56^ The enantiomeric excess of the final product was generally elevated, albeit variable levels of ketone intermediate accumulation were observed (0-32%).

Enantioselective oxidation of secondary alcohols is also feasible using engineered variants of galactose oxidase.^57^

These publications highlight how CD-OS can be a useful strategy for deracemisation of racemic alcohols along with the more established DKR. However, only a limited number of studies have been carried out until to date on CD-OS of alcohols.

#### CD-OS to produce chiral amines

Compared to the corresponding CD-OS of alcohols, CD-OS of amines has attracted more interest in the academic community. The reason may be inferred to the more problematic racemization in DKR approaches for amines compared to alcohols, such as the requirement for harsher conditions that reduce the operational compatibility window with the biocatalysts as illustrated in the DKR section.

**Scheme 9:**
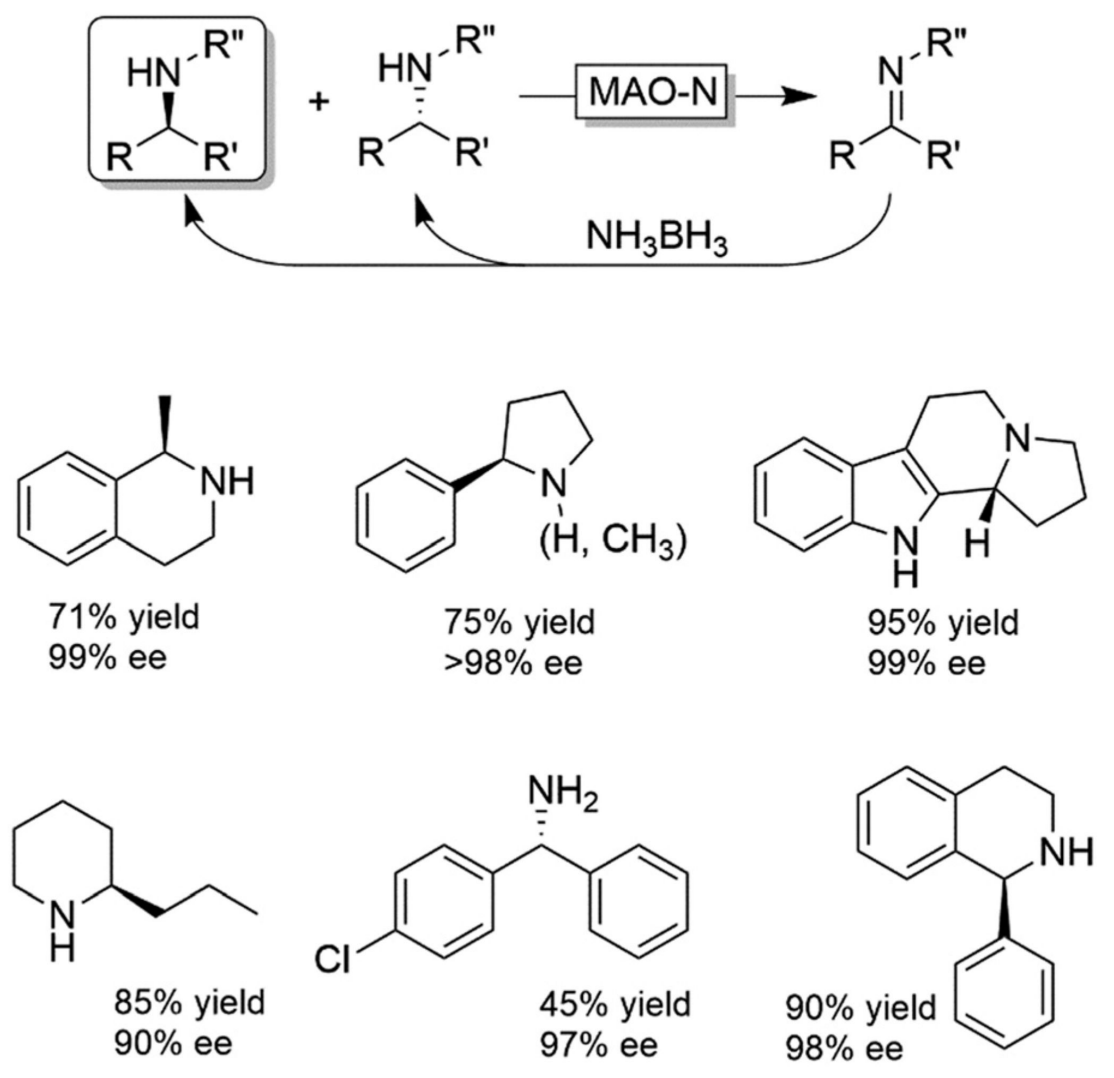
Cyclic deracemisation *via* stereospecific oxidative step (CD-OS) of amines using monoamine oxidase (MAO-N, EC:1.4.3.4) and its variants together with NH_3_BH_3_ as a stoichiometric, non-stereoselective reductant.

Probably the most widespread enzyme used in CD-OS of amines is the monoamine oxidase from *Aspergillus niger* (MAO-N, EC 1.4.3.4) and its manifold mutants.^58,59^ While the wildtype enzyme clearly preferred primary amines, Turner and coworkers have engineered an impressive range of enantioselective variants that exhibit high activities towards pharmaceutically relevant secondary and tertiary amines (e.g., (*R*)-nicotine, crispine A), thus affording excellent yields and often perfect enantiomeric excess.^58^ The same group also introduced ammonia borane (NH_3_BH_3_) as a more versatile reducing agent for the concurrent use with oxidases (Scheme 9).^59^ Today, the combination of MAO-N or its variants and NH_3_BH_3_ has become a standard protocol for the deracemisation of amines into enantiomerically pure amines.^60–64^ A selection of enantiomerically pure amines obtainable *via* the MAO-N-NH_3_BH_3_ strategy is shown in Scheme 9.

However, monoamine oxidases (naturally occurring or engineered) display mainly (*S*)-selectivity towards their substrates, thereby limiting the scope of obtainable enantiomer products. Asano, Turner and their coworkers independently filled this gap by reporting on (*R*)-selective amine oxidases engineered from either a 6-hydroxy-D-nicotine oxidase (6-HDNO)^65^ or a (*R*)-selective amino acid oxidase from porcine kidney.^66^

An interesting alternative to NH_3_BH_3_ as a stoichiometric reductant has been proposed by Lloyd and coworkers.^67^ Whole cells of recombinant *E. coli* overexpressing MAO-N and presenting Pd-nanoparticles at the surface were used in this approach. Unfortunately, both reaction steps (MAO-N-catalysed KR and nano-Pd-catalysed non-stereoselective hydrogenation of the imine intermediate) had to be performed sequentially due to the formation of undesired by-products in the concurrent mode.

**Scheme 10:**
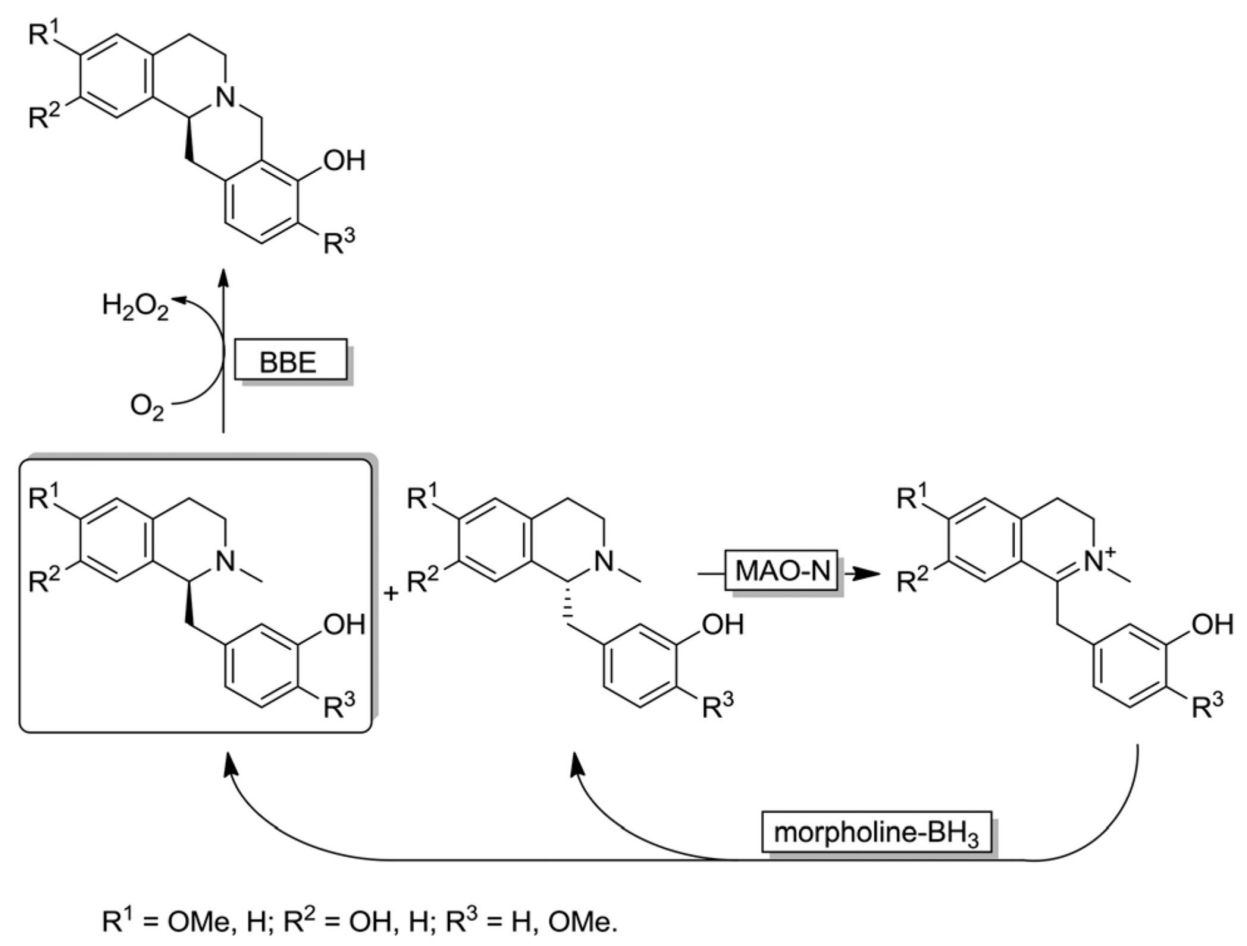
Cyclic deracemisation *via* enantioselective oxidative step (CD-OS) of tetrahydroisoquinoline alkaloids using MAO-N 11 variant and morpholine-borane combined with a further enantioselective oxidative cyclization catalysed by berberine bridge enzyme (BBE).

Interestingly, the MAO-N/BH_3_ system was implemented in a deracemisation by simultaneous bio-oxidative kinetic resolution and stereoinversion. (Scheme 10).^68^ In this study, race-mic 1-benzyl-1,2,3,4-tetrahydroisoquinolines were subjected to cyclic deracemisation by MAO-N/morpholine·BH_3_, while the formed (*S*)-configured tetrahydroisoquinolines were concurrently cyclised by a berberine bridge enzyme (BBE) to yield berberine in elevated yields (79–97%) and enantiomeric excess (>97%). It is noteworthy that DKR of 1-benzyl-1,2,3,4-tetrahydroisoquinolines by a combination of a Shvo’s Ru-catalyst with BBE is inapplicable because the metal catalyst also racemises the final cyclized product.^68^

**Scheme 11:**
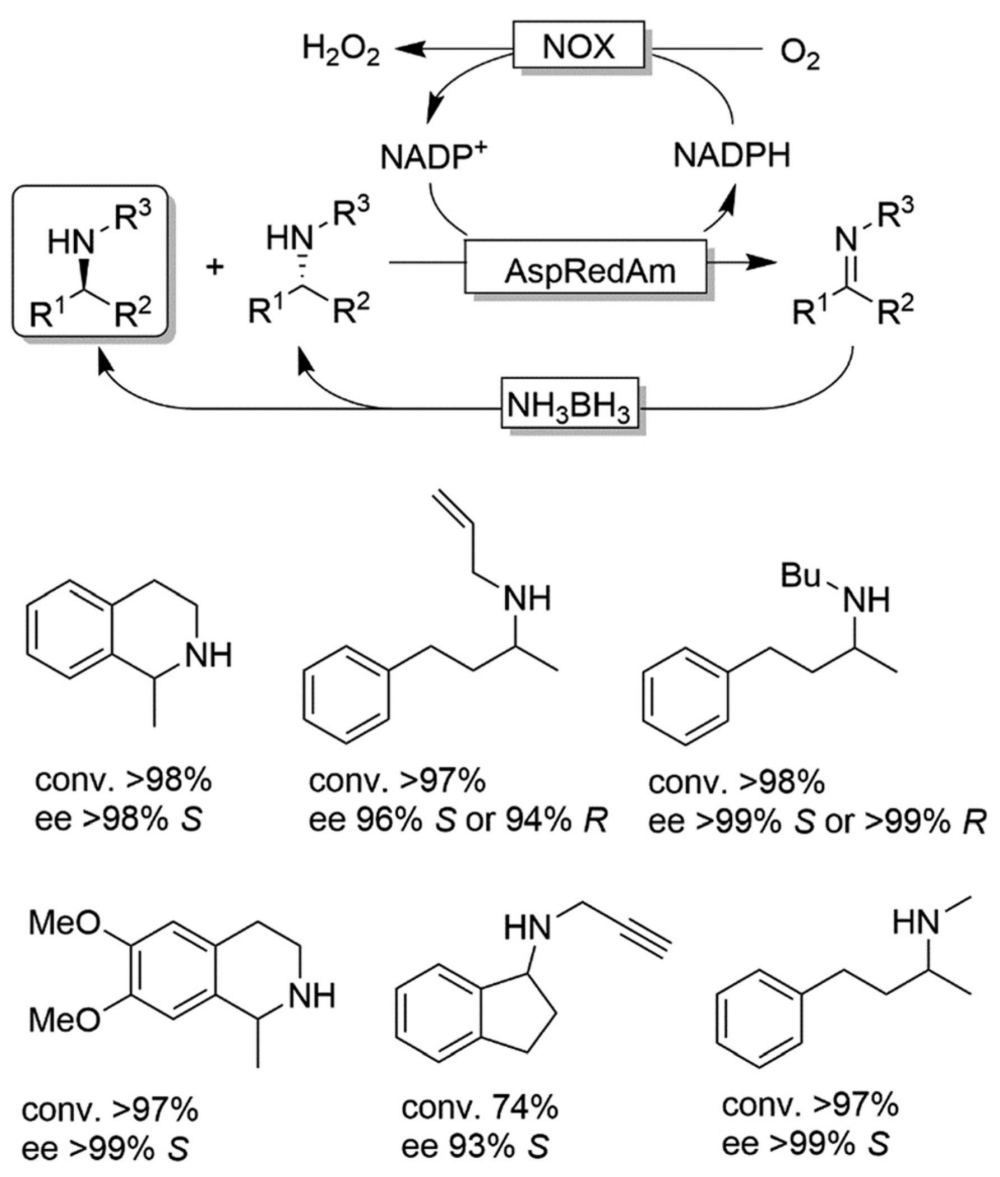
CD-OS of amines exploiting the reversibility of reductive aminases. *In situ* NADP^+^ regeneration was accomplished using NADPH oxidase (NOX). *Asp*RedAm: reductive aminase from *Aspergillus oryzae*.

CD-OS of amines has also been carried out using dehydrogenases rather than oxidases. In fact, a simple KR of primary amines can be accomplished by an amine dehydrogenase (AmDH) in combination with water or H_2_O_2_-forming NAD(P) H oxidase as exemplified by Yun and coworkers.^69^ Therefore, this oxidative deamination can represent the KR part of the CD-OS reaction, thereby overall mimicking the MAO-N system. Very recently, Turner and coworkers reported a CD-OS through the application of a sub-family of imine reductases (called reductive aminases, RedAms) in the KR step of secondary amines (Scheme 11) combined with a reduction step performed by NH_3_BH_3_ as for the MAO system.^70^ It remains to be shown whether this (more complex system) will be able to compete with the very efficient MAO-N system. At the moment, the reported substrate scope is limited to the structures depicted in Scheme 11.

## Linear deracemisation including stereoinversion reactions

**Scheme 12:**
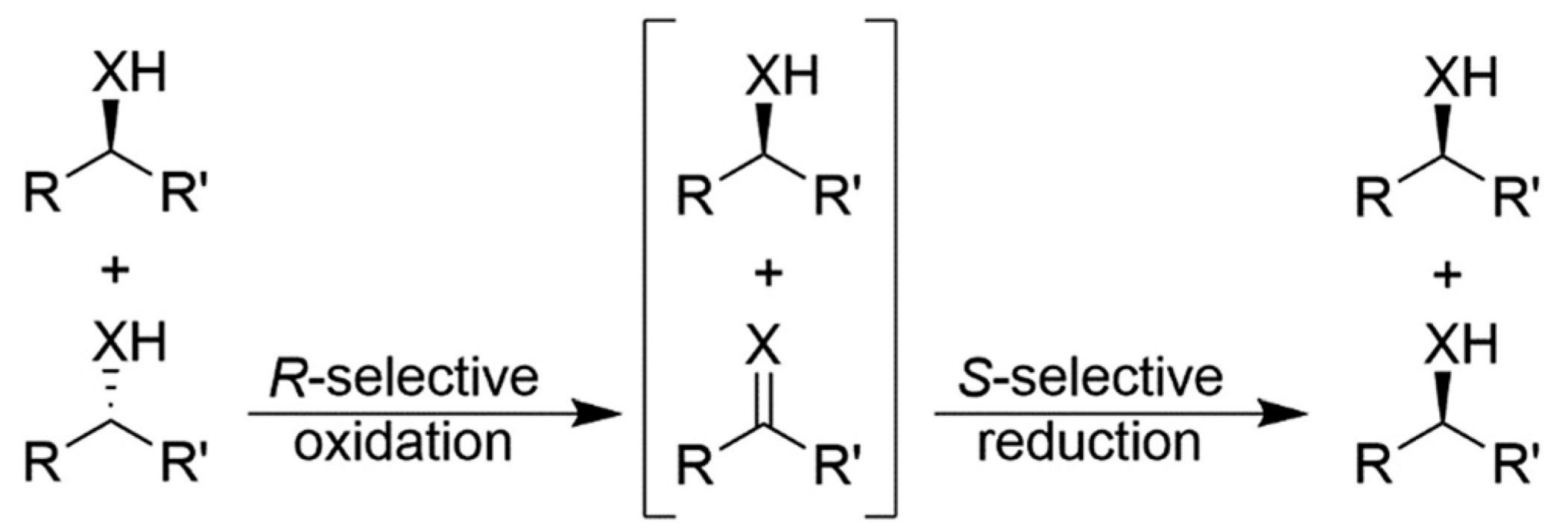
Deracemisation of alcohols (X = O) and amines (X = NH) through stereoinversion, exemplified for deracemisation of a racemic mixture to yield the (*S*)-enantiomer. *R* has a higher Cahn-Ingold-Prelog priority than *R*′.

Stereoinversion reactions resemble the above-described cyclic deracemisation (CD) strategies in the sense that the undesired enantiomer in a first step is oxidised to the prochiral ketone or imine functionality in a theoretically enantioselective manner. Then, the intermediate is reduced stereoselectively to the desired enantiomer (Scheme 12). Hence, as previously observed, stereoinversion-based deracemisations are more efficient compared to CD as only one cycle is necessary. Practically, enzymes exhibiting strict enantiocomplementarity, which are relatively accessible nowadays,^71^ are necessary for stereoinversion reactions.

As suggested by the name, stereoinversion methods can also be used to invert the stereochemistry at a given (enantiomerically pure) alcohol or amine (*i.e*., in this way emulating the outcome of a Mitsunobu-type reaction).

Finally, the same approach can be used for either conversion of racemic alcohols into enantiomerically pure amines or conversion of racemic amines into enantiomerically pure alcohols. These cases will also be discussed in this section.

### Deracemisation of alcohols *via* stereoinversion

#### Concurrent two-step stereoinversion of alcohols (non-separated in time)

**Scheme 13:**
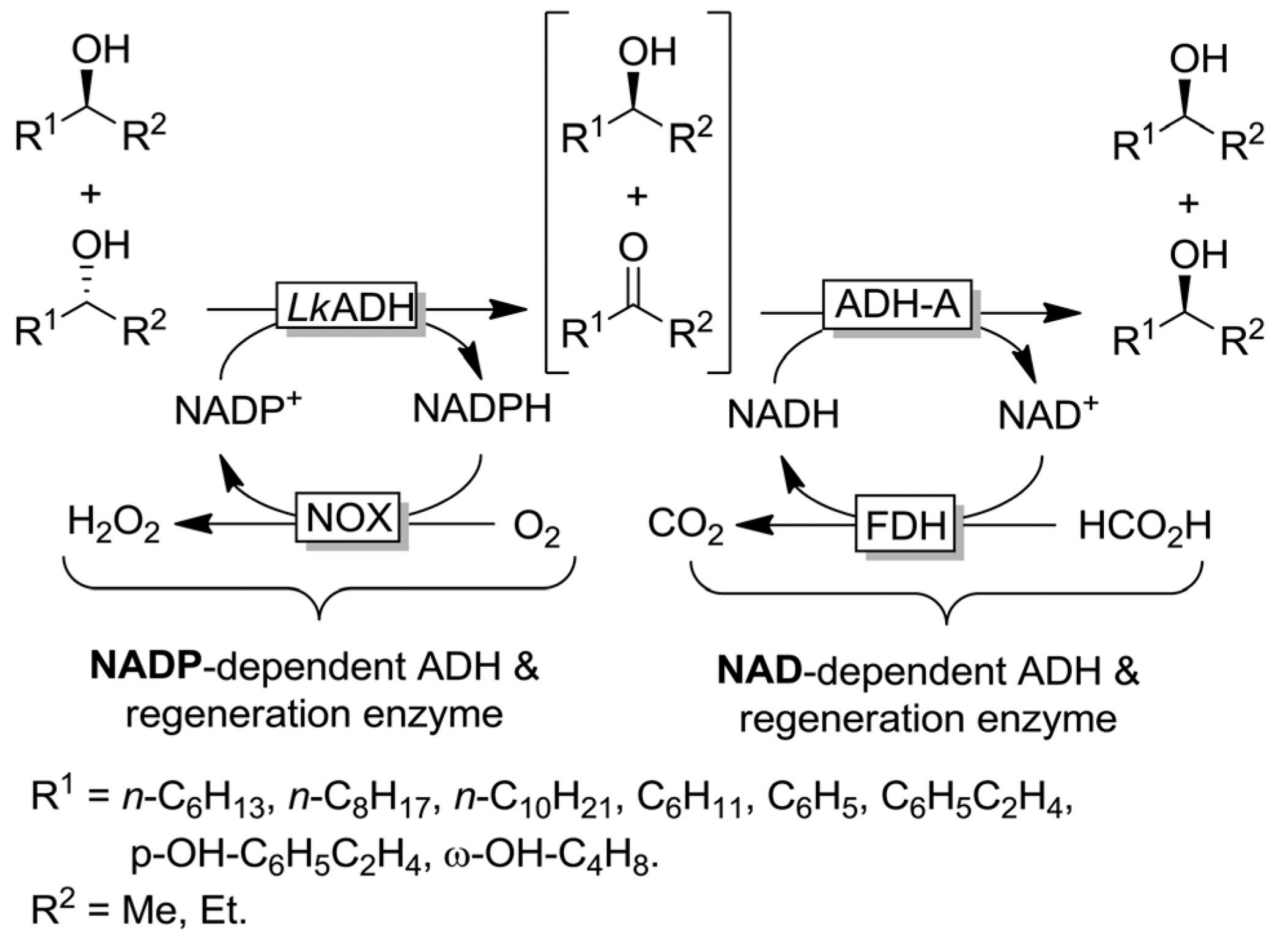
Deracemisation *via* stereoinversion of racemic alcohols combining (*R*)-selective LkADH and (*S*)-selective ADH-A together with the corresponding cofactor regeneration systems. Alcohols with opposite configuration were obtained by combining (*S*)-selective TbADH and (*R*)-selective ADH-PR2. Note: The oxidative and reductive steps are highly enantiocomplementary and orthogonal with respect to co-factor dependency. ADH-A: ADH from *Rhodococcus ruber* (EC 1.1.1.1), LkADH: ADH from *Lactobacillus kefir* (EC 1.1.1.2), FDH: formate dehydrogenase (EC 1.2.1.2), NOX: NADPH oxidase (EC 1.6.3.1).

The deracemisation of alcohols *via* stereoinversion using dehydrogenases in a one-pot and concurrent mode has been pioneered by Kroutil and coworkers.^72^ In this seminal work, the deracemisation of a broad range of race-mic secondary alcohols was attained through a combination of two enantiocomplementary ADHs (Scheme 13). A prerequisite for the realisation of a concurrent process without any physical and temporal separation is that both ADHs must be orthogonal with respect to their cofactor requirements, *i.e*., one ADH must be theoretically strictly NAD-dependent whereas the other one must be strictly NADP-dependent. In practice, the applied ADHs must possess at least 10-fold preference over the desired form of the nicotinamide-adenine dinucleotide cofactor (NADH *vs*. NADPH or *vice versa*).^72^ Both ADHs must be exploited selectively in either reductive or oxidative mode, respectively, in order to avoid futile cycles (*i.e*., which otherwise would lead to a cyclic deracemisation process). This is achieved by concurrent *in situ* regeneration of NADH and NADP^+^ (or NAD^+^ and NADPH).

**Scheme 14:**
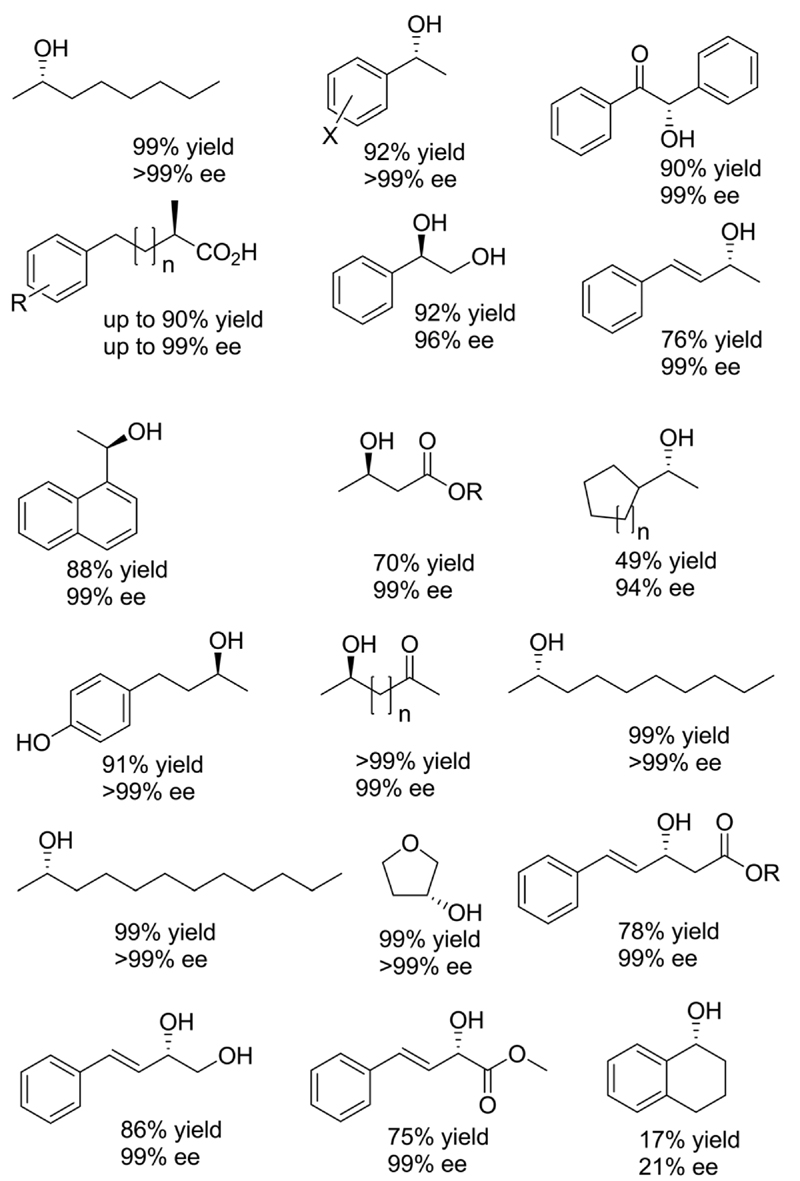
Selection of enantiomerically pure alcohols obtained *via* stereoinversion.^73–81^

The example shown in Scheme 13 yields a broad range of enantiomerically pure (*S*)-configured alcohols. By choosing another combination of enzymes *(e.g.* (*S*)-selective *Tb*ADH and (*R*)-selective ADH-PR2), (*R*)-configured alcohols could be produced as well. Instead of using isolated enzymes, a number of microbial stereoinversion systems as well as hybrid systems containing whole cells/isolated enzymes have also been reported in deracemisation *via* stereoinversion.^73–81^ Here, it should, however, be mentioned that due to the presence of various endogenous ADHs and alcohol oxidases (AOxs) in the microbial “black-box” systems, the real deracemisation pathway may differ case by case from the depiction of Scheme 13; consequently, identifying suitable enantiocomplementary systems can be challenging. Scheme 14 depicts a selection of enantiomerically pure alcohols obtained through enzymatic and microbial deracemisation *via* stereoinversion.

#### Sequential two-step stereoinversion of alcohols *(i.e.* separated in time)

**Scheme 15:**
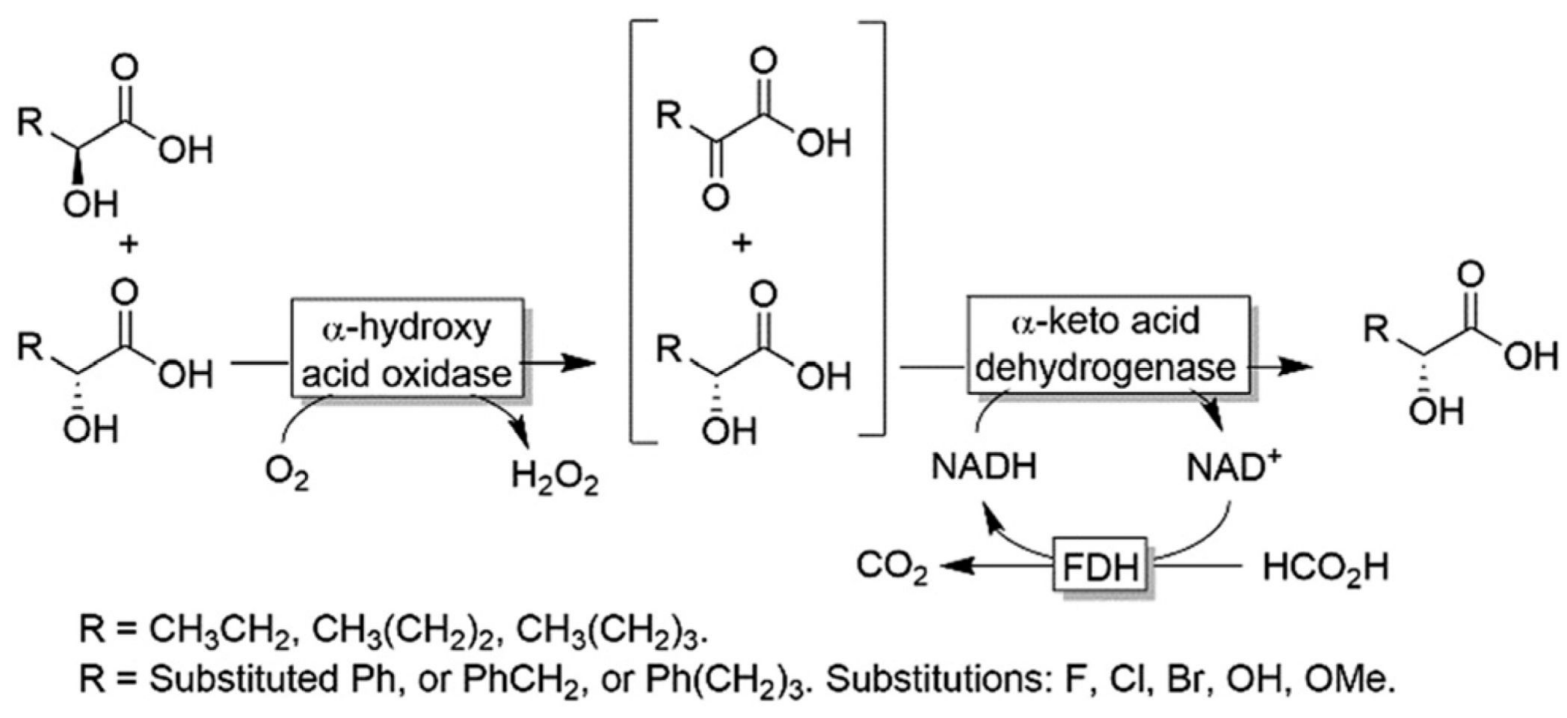
Deracemisation of α-hydroxy carboxylic acids by sequential aerobic oxidation followed by stereoselective reduction.

In 1998, Adam and coworkers reported the deracemisation of racemic 2-hydroxy-carboxylic acids through the combination of a glycolate oxidase and a D-lactate dehydrogenase.^82^ Glycolate oxidase catalysed the stereospecific oxidation of the (*S*)-configured substrate, whereas D-lactate dehydrogenase catalysed the stereoselective reduction of the α-keto carboxylic acid intermediate. The deracemisation could only be accomplished by sequential addition of D-lactate dehydrogenase upon completion of the first oxidative step; in the second step, the NADH cofactor was recycled by the FDH/formate system (Scheme 15). The one-pot approach performed fairly well with aliphatic substrates (84-93% yield, 67-91% ee, *R)* but failed with aryl-containing substrates. The concept was recently reinvestigated by using alternative α-hydroxy carboxylic acid oxidases and α-keto carboxylic acid dehydrogenases^83^ Thus, the substrate scope of the reaction could be extended to include phenyl as well as aryl-aliphatic α-hydroxy carboxylic acids with variable conversions (43-98%) but excellent enantiomeric excess (>99% *R* in most cases).

**Scheme 16:**
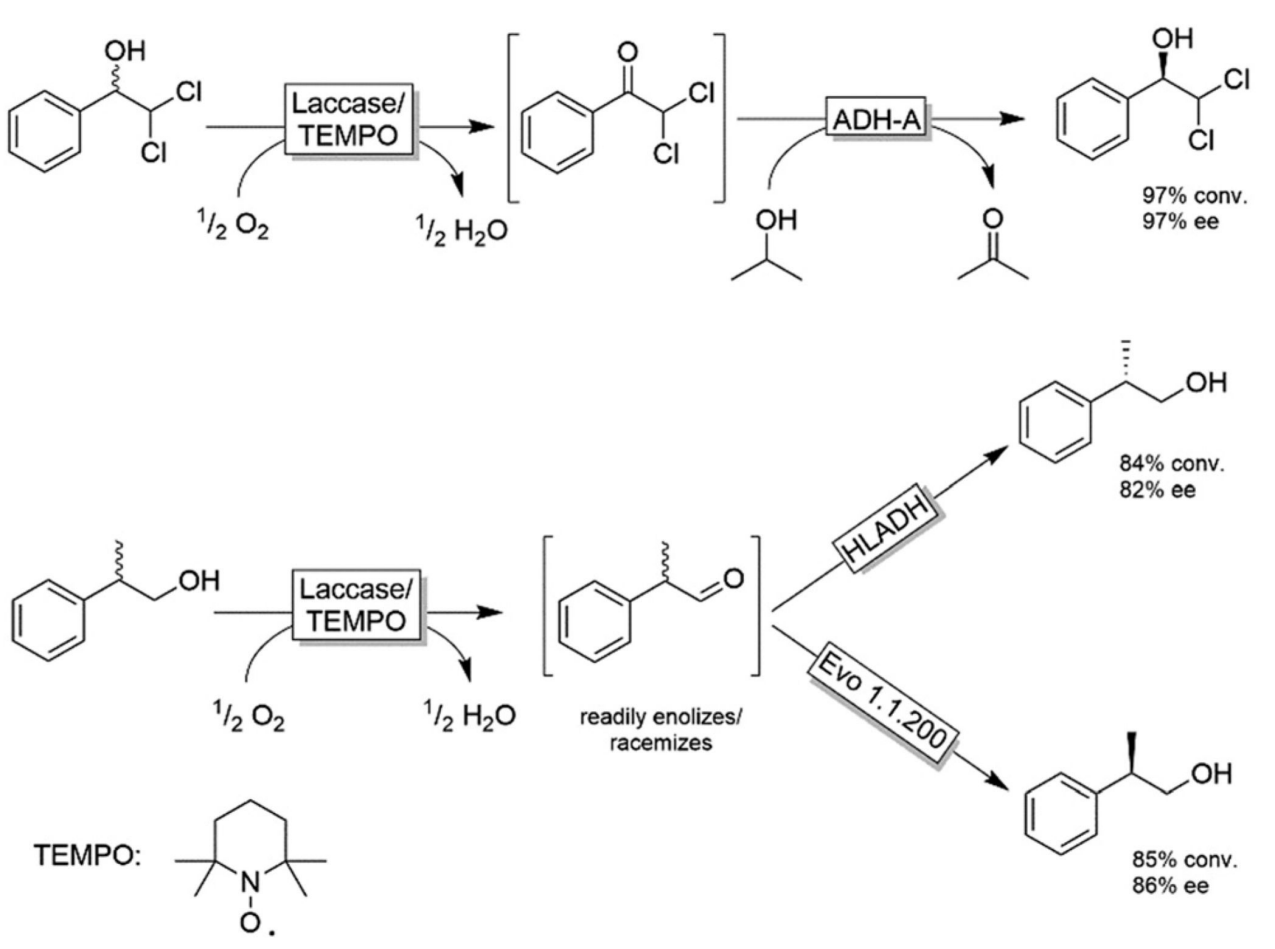
Examples for deracemisation using the laccase mediator system. ADH-A: ADH from *Rhodococcus ruber* (EC 1.1.1.1), HLADH: horse liver ADH (EC 1.1.1.1), Laccase from *Trametes versicolor* (EC 1.10.3.2), TEMPO: (2,2,6,6-tetramethylpiperidin-1-yl)oxidanyl.

The extension of this system to substrates devoid of the α-carboxylic moiety was explored by Gotor, Gotor-Fernandez and coworkers (Scheme 16).^84–86^ They applied either TEMPO/I_2_ or the established laccase-mediator system (LMS) for the non-stereoselective oxidation of primary and secondary alcohols followed by a second stereoselective reduction step of the intermediate carbonyl group. In all these studies, the reduction was catalysed by an ADH, either as whole cell or lyophilized crude biocatalyst. Scheme 16 depicts selected examples for this concept. The TEMPO-I_2_/*E*. *coli*(ADH) chemo-enzymatic sequential system was applied for the deracemisation of 1-phenylethanol as well as selected aliphatic alcohols to yield enantiomerically pure alcohols (ee >99%) in nearly quantitative conversion.^84^ The use of stereocomplementary ADHs enables the isolation of either *(R)-* or (*S*)-configured alcohols. However, the overall deracemisation required 2 equiv. of I_2_ as a sacrificial oxidant along with 0.2 equiv. of TEMPO as the oxidation catalyst, whereas the enzymatic reduction step was driven to completion by using an excess of 2-propanol as a cosubstrate. The laccase/TEMPO system was successfully coupled to *E*. *coli*(ADH) cells in the deracemisation of aromatic α-dichloro alcohols as model substrates to afford the final product with variable levels of yields (62–95%) and elevated ee (97%).^86^ The advantage of the laccase/TEMPO compared to the TEMPO-I_2_ systems in the oxidation step is that the former consumes only dioxygen as oxidant.

In the case of deracemisation of 2-phenyl-1-propanols, the laccase/TEMPO oxidation generates an enolizable intermediate under the reaction conditions; thus, by using stereocomplementary ADHs, both enantiomers could be obtained in high yields (95% or higher) but moderate optical purities in most cases (6-94%).^85^ A variation of this method using 2-azaadamantane N-oxyl as oxidation catalyst and HOCl as a stoichiometric oxidant was reported recently.^87^

**Scheme 17:**
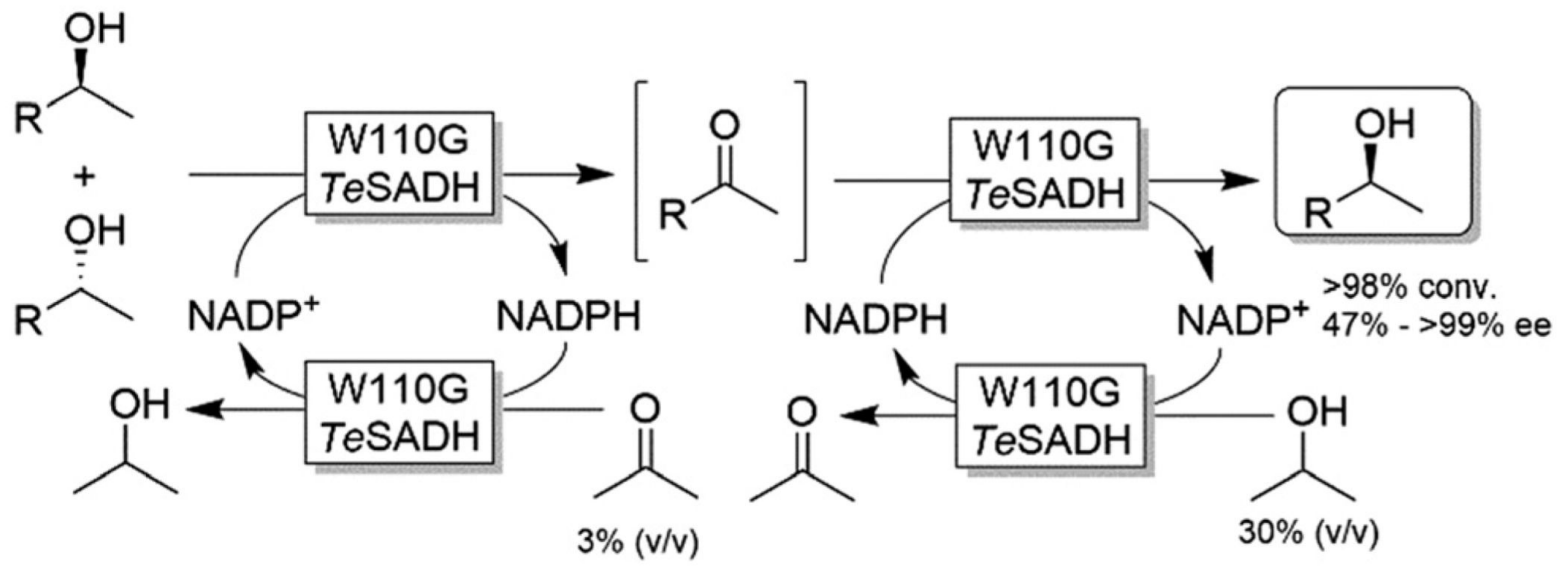
One-pot two-step deracemisation of secondary alcohols using a single enzymatic approach. *Te*SADH: *Thermoanaerobacter ethanolicus* secondary ADH (EC 1.1.1.2).

Musa and coworkers reported a single enzymatic two-step deracemisation approach of secondary alcohols using W110G *Te*SADH (Scheme 17).^88^ It relies on facilitating “selectivity mistakes” in the oxidation pathway by applying a precise concentration of acetone cosubstrate; this approach enables the depletion of both enantiomers of an alcohol racemate to afford the corresponding prochiral ketone, the latter of which is subsequently reduced—under stereoselective conditions and using the proper concentration of 2-propanol—to the corresponding (*S*)-configured alcohol. Thus, the overall reaction proceeds in one-pot removing the need to isolate the ketone intermediate. More recently, the same strategy was implemented in stereoinversion of (*R*)-configured alcohols (*i.e*., mimicking the actions of Mitsunobu reaction).^89^

### Linear deracemisation of amines *via* stereoinversion

**Scheme 18:**
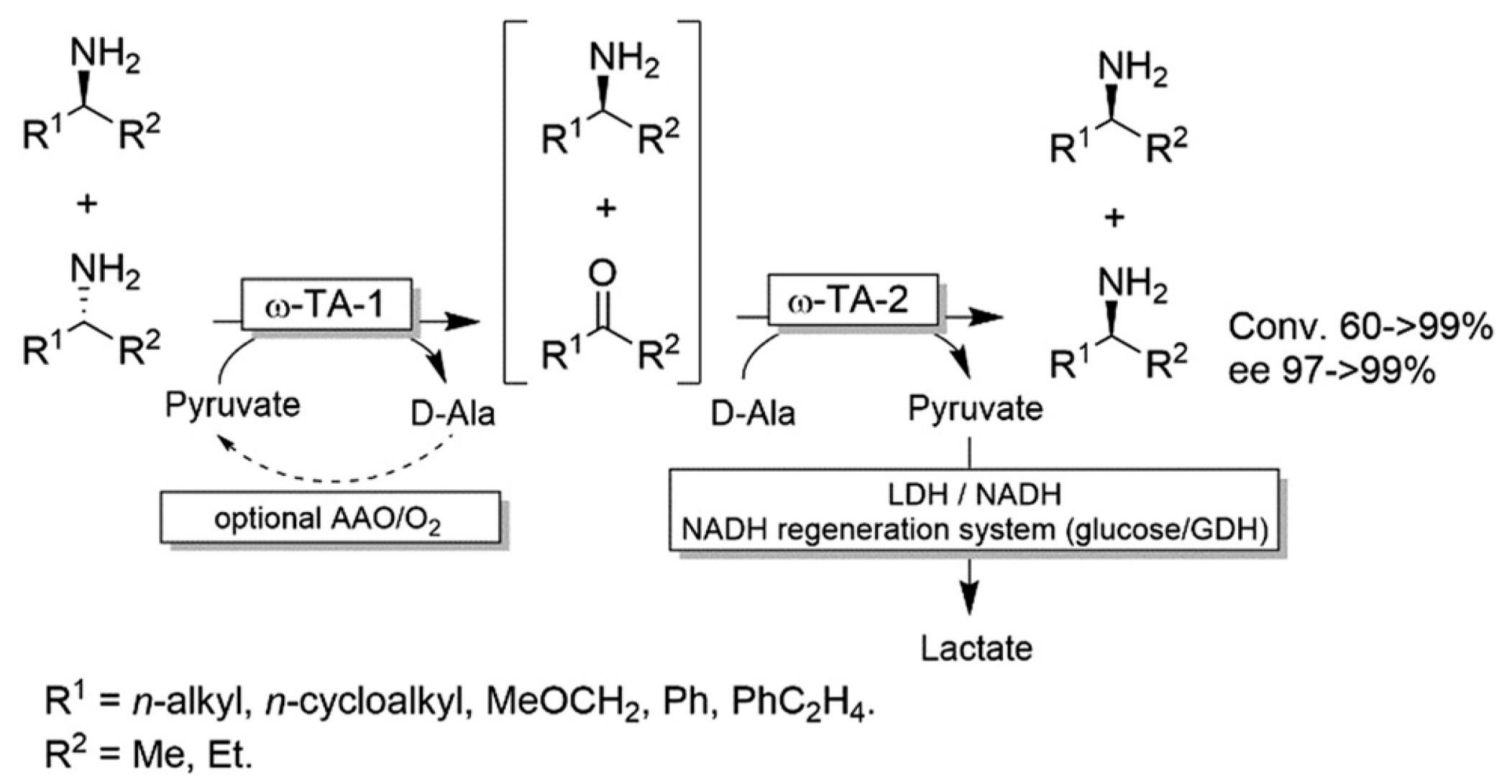
One-pot deracemisation of amines using enantiocomplementary ω-TAs in two temporally separated steps. LDH: Lactate dehydrogenase (EC 1.1.1.27), AAO: amino acid oxidase (EC 1.4.3.2), GDH: glucose dehydrogenase.

For stereoinversion of amines nowadays a broad range of enantiocomplementary ω-transaminases (ω-TAs) are available. The major disadvantage of these methods, however, is that no orthogonal systems are available today. Accordingly, ω-TA-based stereoinversions of amines can be performed in one-pot, albeit in two steps separated in time; thus, the general protocol often includes the inactivation of the first ω-TA used (*i.e*., employed in the KR step) to avoid re-formation of the undesired enantiomer in the second formal reductive amination step operated by the stereocomplementary enzyme (Scheme 18).^90^ In this initial work, a stoichiometric amount of pyruvate was consumed in the first KR step, whereas the second amination step required the addition of further five equivalents of alanine along with a bi-enzymatic pyruvate-removal network (*i.e*., NAD (cat.)/LDH/GDH/glucose (*ca*. 3 equiv.)) in order to shift the thermodynamic equilibrium towards amine formation. Notably, the atom-efficiency of the deracemisation was improved by performing the first KR step using a catalytic amount of pyruvate, which was recycled *in situ* by an amino acid oxidase (AAO) at the expense of dioxygen. The strategy was applied to the deracemisation of the drug Mexiletine in hundred milligram-scale (97% yield, ee >99% *S* or *R*).^90^ In all these studies, the first ω-TA used for KR was thermally deactivated before starting the second step. In a follow-up study, immobilised enzymes were employed thereby enabling a more convenient removal of the ω-TA used in the first step.^91^

**Scheme 19:**
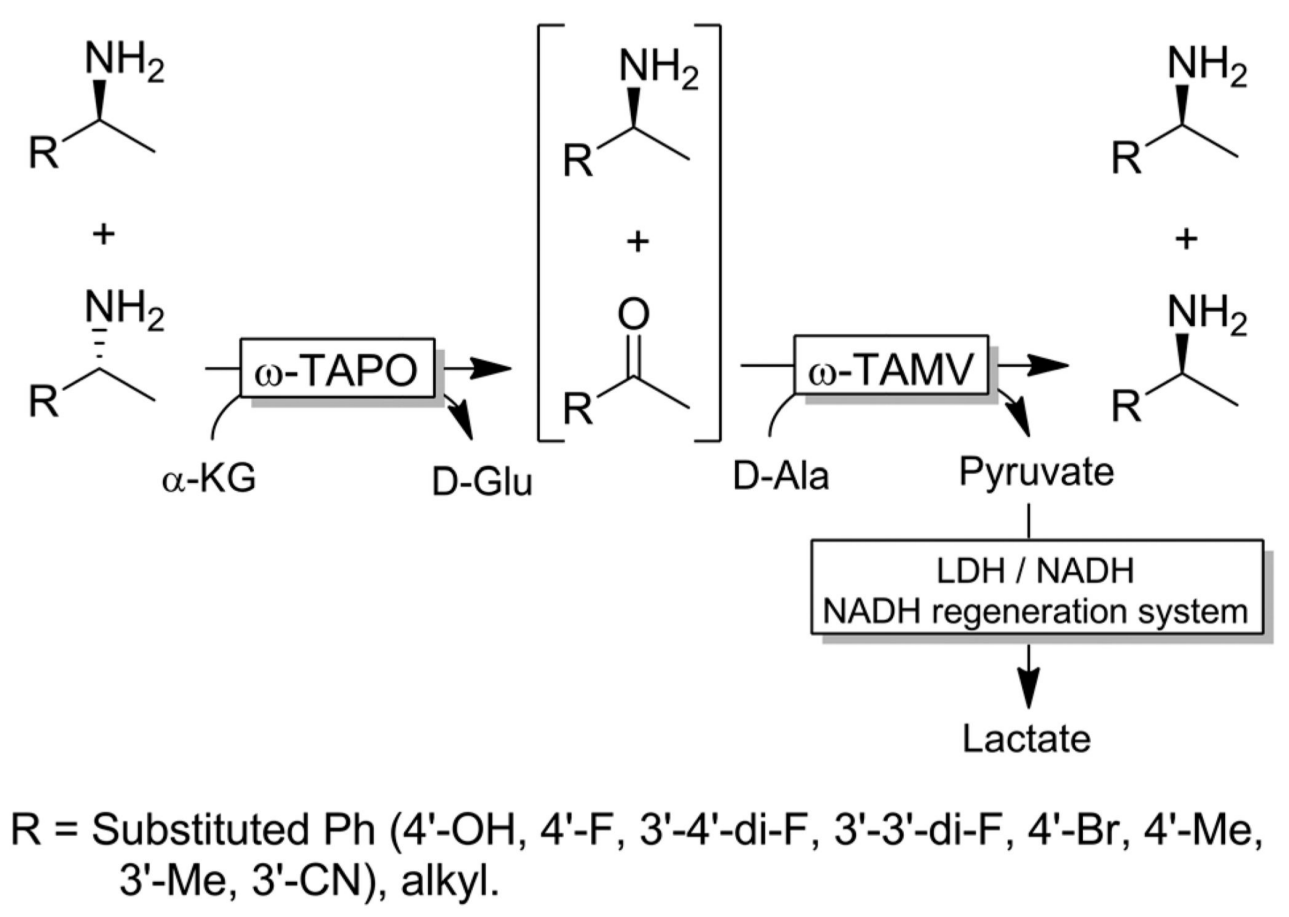
One pot deracemisation *via* stereoinversion of amines using two orthogonal (with respect to the preferred amine-donor or acceptor) and enantiocomplementary ω-TAs. TAPO: ω-transaminase from *Polaromonas* sp., TAMV: ω-transaminase from *Mycobacterium vanbaalenii* (EC 2.6.1.18), LDH: lactate dehydrogenase (EC 1.1.1.27).

Yun and coworkers presented a possible solution to circumvent the bioorthogonality issue by introducing a new ω-TA from Polaromonas sp. (ω-TAPO) that is capable of accepting 2-ketoglutarate as a sacrificial cosubstrate (thus yielding glutamic acid as a by-product).^92^ In combination with one other ω-TA from a selected pool (*e.g*., from *Mycobacterium vanbaalenii*, ω-TAMV), which did not accept aspartic acid as an amine donor, oxidative deamination KR reaction could be performed simultaneously with the stereoselective formal reductive amination reaction (Scheme 19). This approach was improved further by using a (R)-selective ω-TA from *Arthrobacter* sp. (ARTA) a (*S*)-selective ω-TA from *Ochrobactrum anthropic* (OATA) and using pyruvate and iso-propyl amine as cosubstrates.^93^ This approach has the advantage of eliminating the use of the expensive 2-ketoglutarate and D-alanine cosubstrates and having acetone as a coproduct, which can be removed under reduced pressure. Certainly, this approach will receive more attention in the future.

**Scheme 20:**
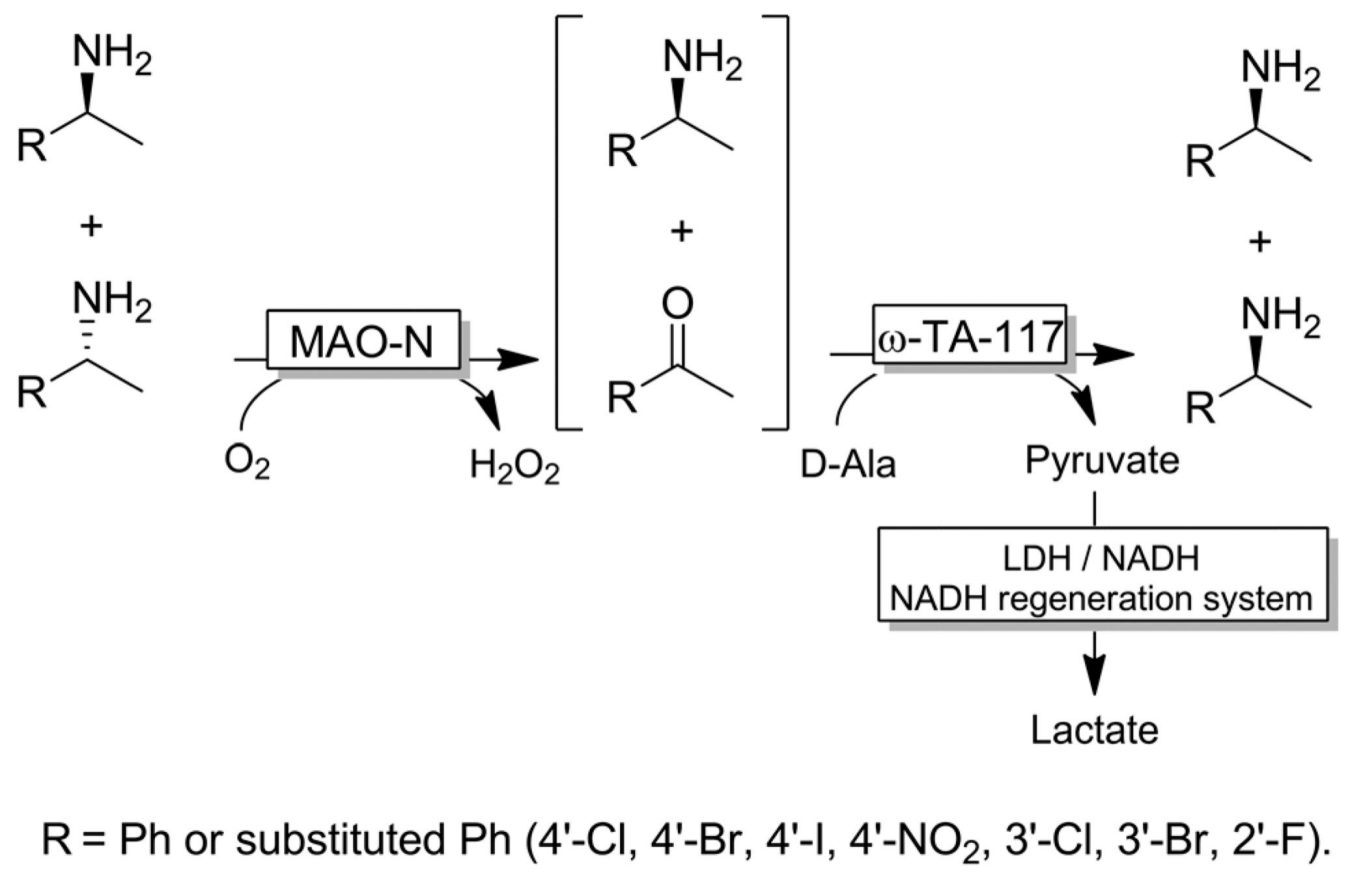
Deracemisation of amines combining MAO-N-catalysed KR with ω-TA-catalysed reductive amination. MAO-N: Monoamine oxi-dase from *Aspergillus niger* (MAO-N, EC:1.4.3.4), LDH: lactate dehydrogenase (EC 1.1.1.27).

Besides ω-TAs, amine oxidases can be used for the first step (*i.e*., KR) of the stereoinversion cascade (Scheme 20).^94^ In this case, provided that the enzymes used are highly stereoselective, no cross-reactivities or futile cycles occur. However, the substrate scope of the reaction was limited to substituted 1-benzyl-ethylamines because of the intrinsic catalytic activity of MAO-N.

**Scheme 21:**
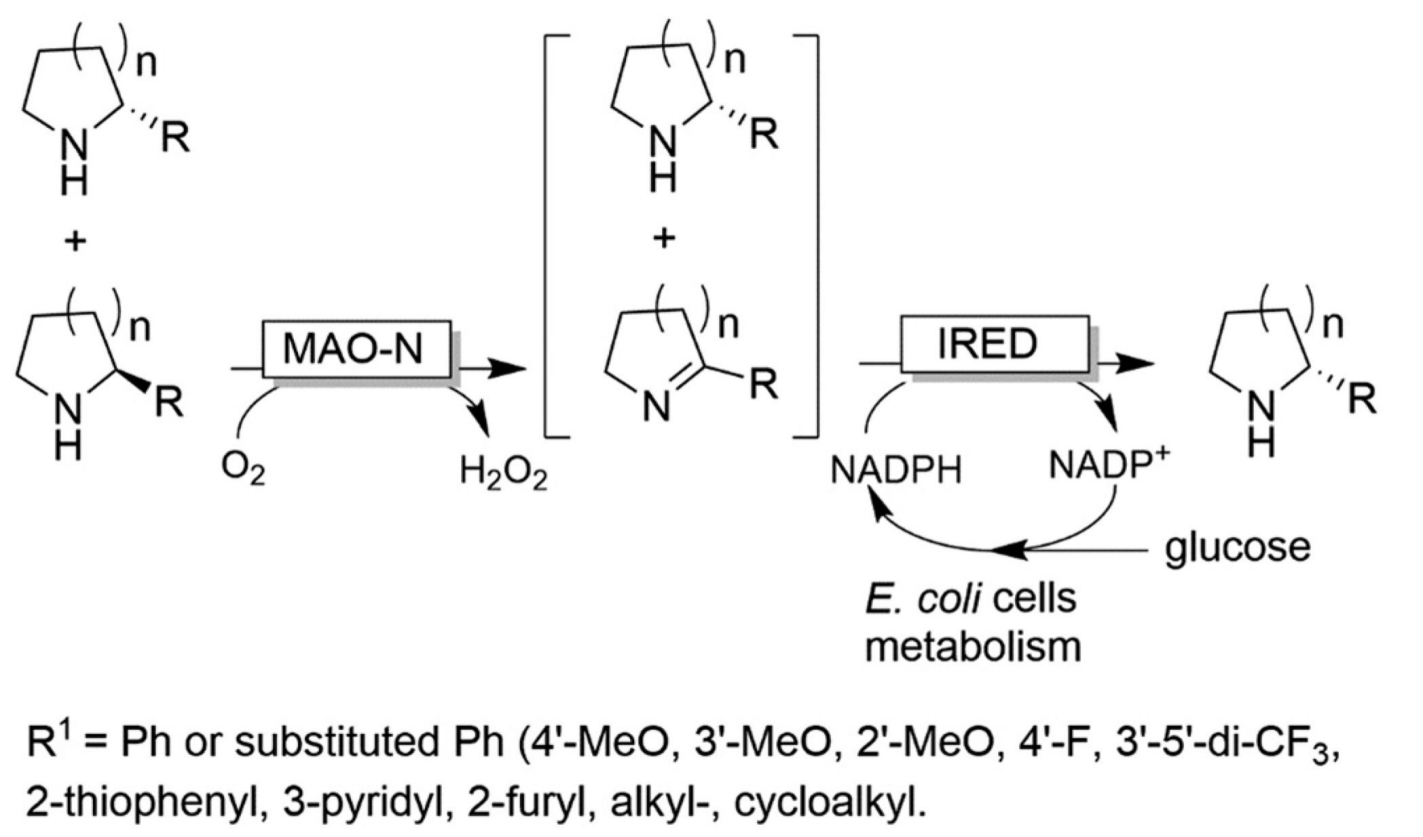
Simultaneous one-pot deracemisation of secondary amines by combining monoamine oxidases (MAO-N) and imine reduc-tases (IRED). The biocatalysts were expressed separately and used as resting *E*. *coli* cells.

In a similar approach (albeit using amino acid oxidases), homoalanine was deracemised.^95^ Finally, it is worth mentioning that the stereoselective amination can also be accomplished either by dehydrogenases such as imine reductases (IRED),^96^ or artificial transfer hydrogenases^97^ rather than ω-TAs. The advantage of this system stems from the possibility to also deracemise secondary amines. In particular, the MAO-N/IRED system was implemented using resting *E*. *coli* cells overexpressing the enzymes separately; thus, the *E*. *coli* metabolism could be exploited for NADH regeneration (Scheme 21). In another work, para-bromo-L-phenylalanine was converted into the opposite enantiomer by performing a one-pot enantioselective oxidative deamination catalysed by an amino acid dehydrogenase (AADH) followed by a formal stereoselective reductive amination catalysed by a α-TA.^98^

**Scheme 22:**
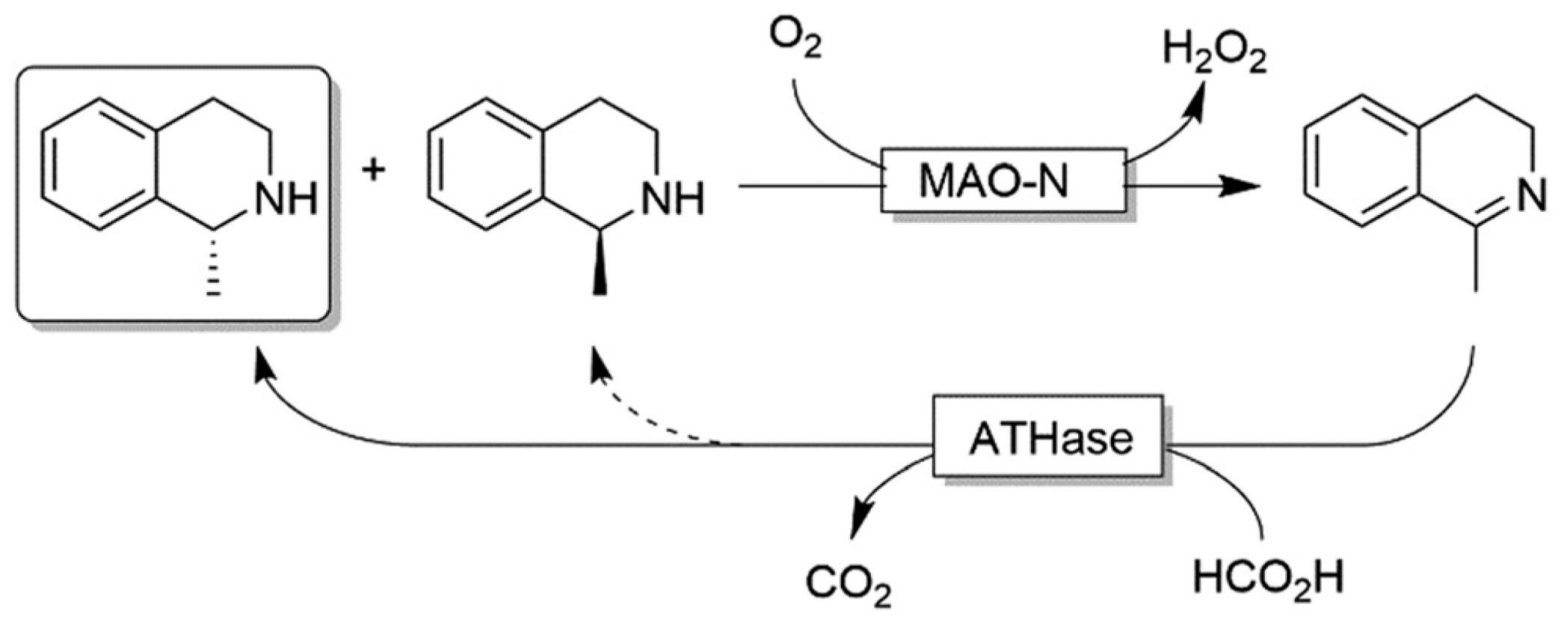
Chemo-enzymatic deracemisation of secondary amines combining MAO-N with an artificial Ir-based enzyme.

Ward and coworkers reported a chemo-enzymatic stereoinversion (*i.e*., CD-ORS) of secondary amines (Scheme 22) by combining MAO-N with an artificial transfer hydrogenase (ATHase).^97^ The oxidative step is the classical MAO-N-based oxidation at the expense of dioxygen; the second step is a stereoselective imine reduction that is performed by an artificial enzymes in which an iridacycle organometallic-catalyst is embedded in a protein scaffold, thus providing the stereo-selective environment. Notably, the reduction step required simple formate as sacrificial reductant. However, as the stereoselectivity of the ATHase is not always perfect, more than one cycle can be required to reach full deracemisation.

### Linear deracemisation of alcohols to optically active amines

**Scheme 23:**
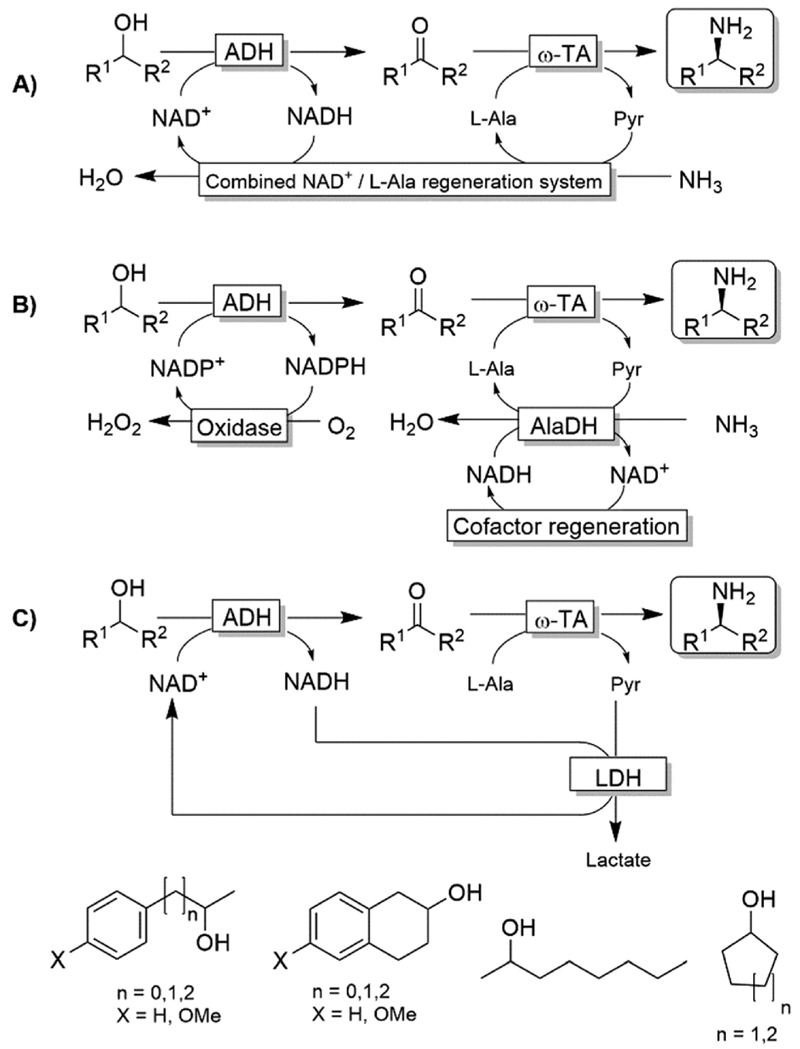
Redox-neutral systems emulating an aza-Mitsunobu reaction comprising ADH-catalysed oxidation of an alcohol followed by the ω-TA-catalysed reductive amination. A) The combined NAD^+^/l-Ala regeneration system comprises l-AlaDH-catalysed reductive amination of pyruvic acid. l-AlaDH: l-alanine dehydrogenase (EC 1.4.1.1). B) Variation of the system comprising orthogonal oxidation and amination steps. C) Variation of the system with pyruvate-removal coupled with NAD^+^-regeneration. Although only system C generates a stoichiomet-ric amount of lactate as waste, all the systems require a supra-stoichiometric amount of alanine as sacrificial amine donor.

Kroutil and coworkers generated a multi-enzymatic network that combined the oxidation of alcohols to their corresponding carbonyl compounds—catalysed by an ADH—to a subsequent formal reductive amination of the intermediate, which is catalyzed by a ω-TA (Scheme 23a).^99,100^ The system was initially applied to the synthesis of non-chiral amines and diamines starting from primary alcohols and diols. This redox-neutral alcohol amination network comprises an l-alanine dehydrogenase (l-AlaDH)-catalysed simultaneous regeneration of both NADH and amine-donor (l-alanine); thus, it produces theoretically only water and consumes ammonia. However, besides the requirement for three enzymes (ADH, ω-TA and l-AlaDH) and two cofactors (NAD^+^ and pyridoxal 5′-phosphate (PLP)), five equivalents of l-alanine as an amine donor were required. In another independent study, Janssen and co-workers could run the same system for the amination of primary ether-alcohols (10 mM) using a catalytic amount of l-alanine as the amine donor (5 mol%); however, the maximum conversion into the amine was only 30%.^101^ These results indicated that an excess of sacrificial amine donor might be mandatory in order to achieve a significant amine product formation with this system. In this context, the deracemisation-amination of secondary alcohols (50 mM) by the ADH/ω-TA/ l-AlaDH system proved to be even more challenging (Scheme 23a).^99^ In fact, the amination led to moderate levels of amine formation (10–64%) along with a significant accumulation of ketone intermediates (up to 47%). The use of orthogonal oxidation (NADP) and amination (l-Ala/NAD) regeneration systems afforded a maximum conversion of 24% (Scheme 23b), whereas pyruvate removal coupled with NAD regeneration afforded a maximum of 64% conversion and 96% ee (Scheme 23c). Applying the system depicted in Scheme 23a, Skerra and coworkers observed an analogous trend in the enantioselective mono-amination of renewable isosorbide (200 mM concentration) that proceeded with 7% conversion, but excellent selectivity (d.e. <99%).^102^

**Scheme 24:**
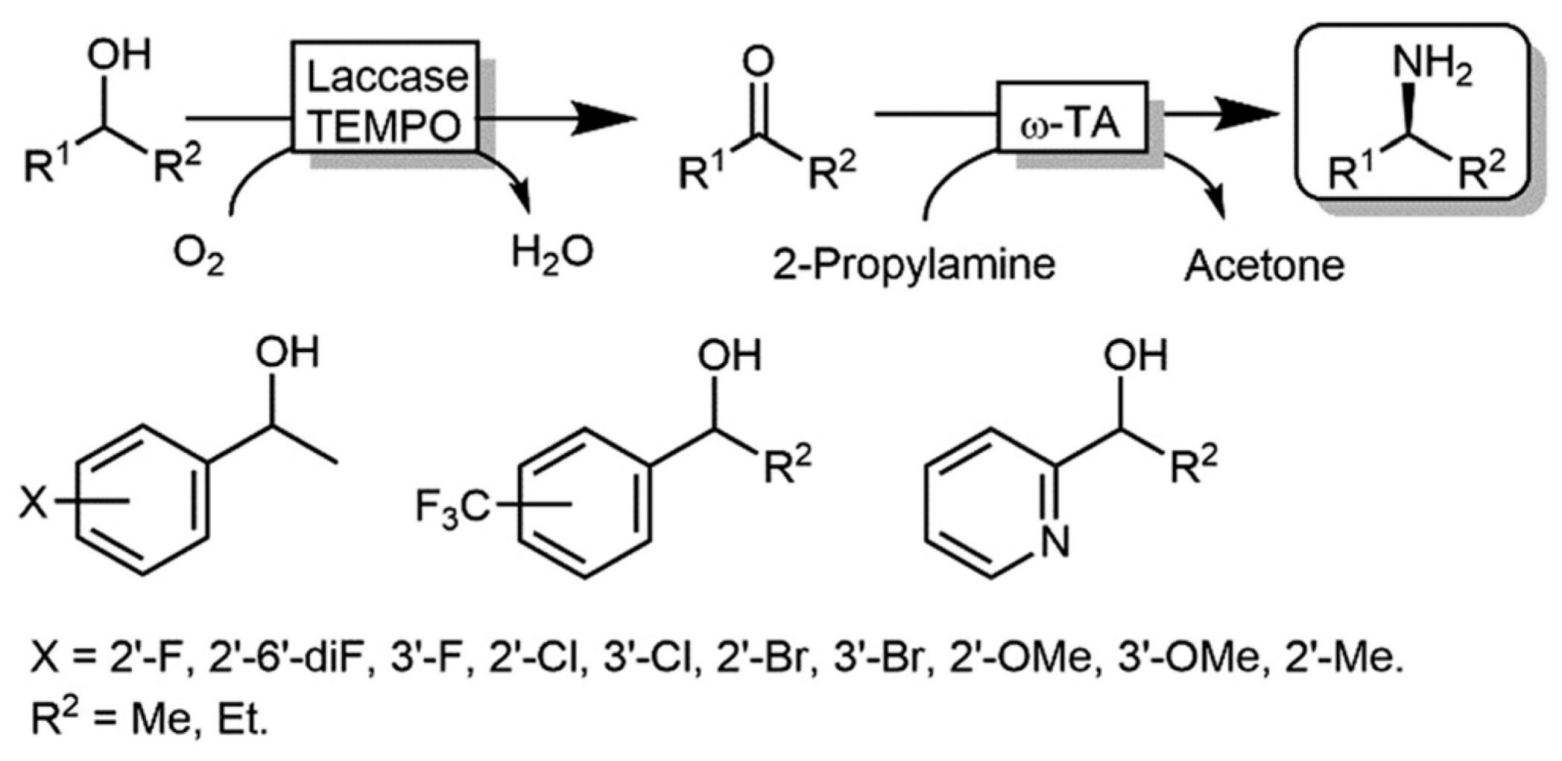
Deracemisation of alcohols to yield optically pure amines by combining laccase-TEMPO oxidation and reductive amination mediated by ω-TAs.

The deracemisation of alcohols to optically active amines is, in principle, possible by combination of an oxidative step catalysed by an alcohol oxidase (AOx) with a concurrent amination catalysed by a ω-TA. In reality, the apparent scarcity of AOxs that are able to oxidize racemic mixtures of secondary alcohols to their ketone intermediates, has limited this strategy to the amination of primary alcohols until to date.^103,104^ Gotor and coworkers overcame this limitation by applying the aforementioned laccase/TEMPO system in the oxidative step, in this case in combination with the ω-TA-catalysed amination (Scheme 24).^105^ Additionally, this latter system exploited the capability of a number of commercially available ω-TAs, to accept 2-propylamine as a sacrificial amine donor. The deracemisation-amination on 50 mM alcohol concentration yielded conversions from 67% to >99%, using 1.33 M of 2-propylamine to push the equilibrium into the desired direction. Notably, the final amine product was obtained in high enantiomeric purity (90 ≥ 99%).

**Scheme 25:**
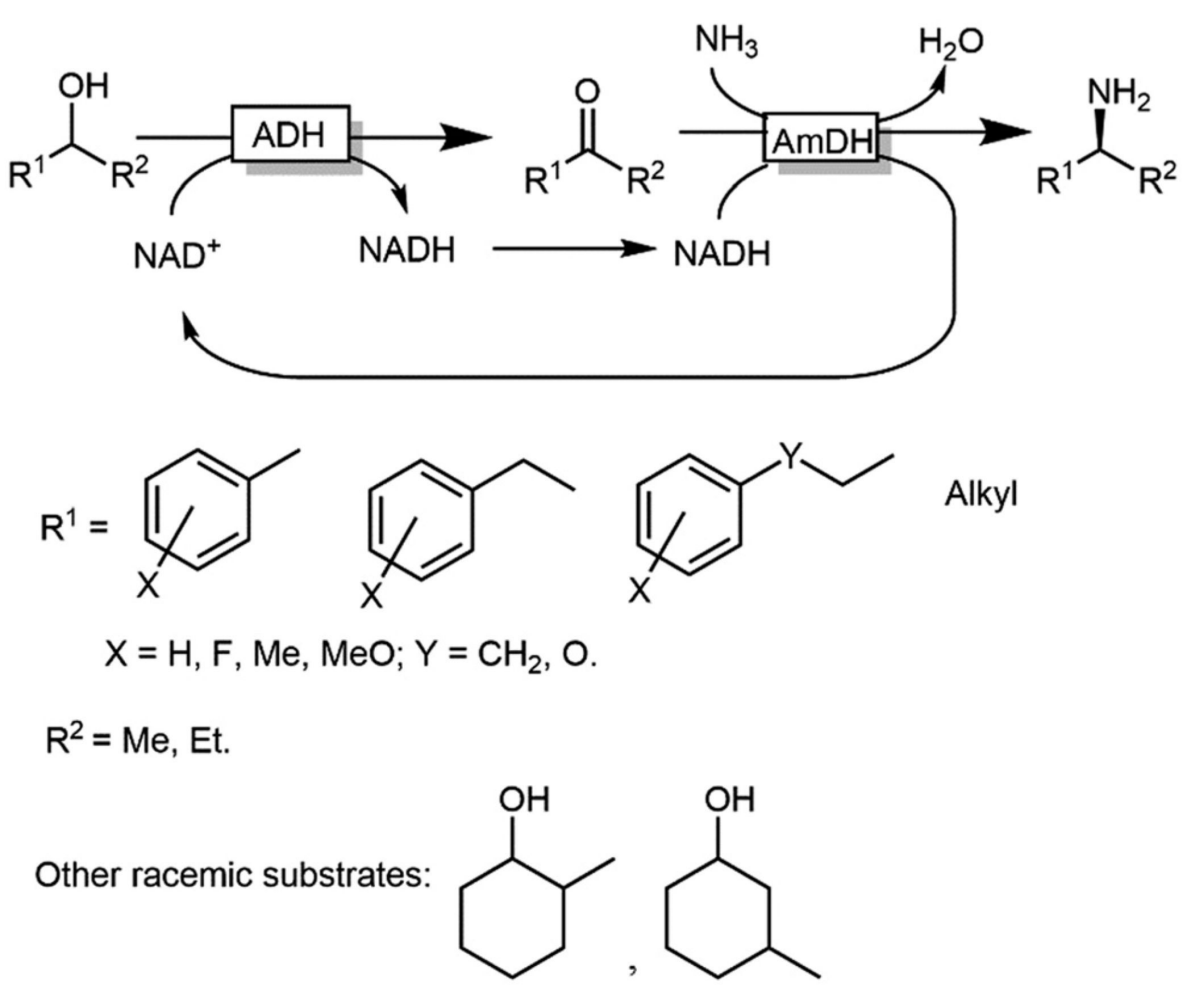
Hydrogen borrowing cascade for the transformation of alcohols into primary and secondary amines combining ADHs and AmDHs.

ω-Transaminases bear the intrinsic disadvantage of relying on organic amines as N-donors for the reductive amination reaction. Therefore, issues of low atom efficiency and kinetic impediments such as product inhibition limit the applicability of these ω-TA-based cascades. More recently, however, amine dehydrogenases (AmDHs) and imine reductases (IREDs) dramatically expanded the scope of the formal bio-catalytic aza-Mitsunobu reactions.^106–111^ If combined with an ADH-catalysed alcohol oxidations, AmDHs enable the direct use of NH_3_ as N-donor (Scheme 25). In this hydrogen-borrowing cascade, the hydride abstracted from the alcohol is re-introduced in the reductive amination step. In general, two ADHs with opposite stereopreferences have been commonly used together in the oxidation step,^106^ depending on the substrate of choice; conversions varied from 9% to 96% while enantiomeric excess was excellent (ee > 99%, *R)* in most of the cases. Attempts to employ a single ADH possessing low enantioselectivity in the oxidation step provided modest results until to date, since a significant amount of alcohol substrates was detected at the end of the reaction (as average *ca.* 30%, and up to 82%), besides ketone accumulation (5-15%).^110^ Unfortunately, the enantiomeric excess of the remaining alcohol substrate was not determined in this study, which complicates the rationalisation of the incomplete conversion to amine; however, it is likely that the incomplete alcohol oxidation in several cases stems from the retained enantioselectivity of the used ADH variant (W110A/G198D *Te*SADH, an NAD^+^-dependent *Te*SADH), which prevents the complete depletion of the both alcohol enantiomers. Thus, engineering or discovery of non-enantioselective ADHs that are applicable on a wide range of substrates still represents a major challenge in this area.

**Scheme 26:**
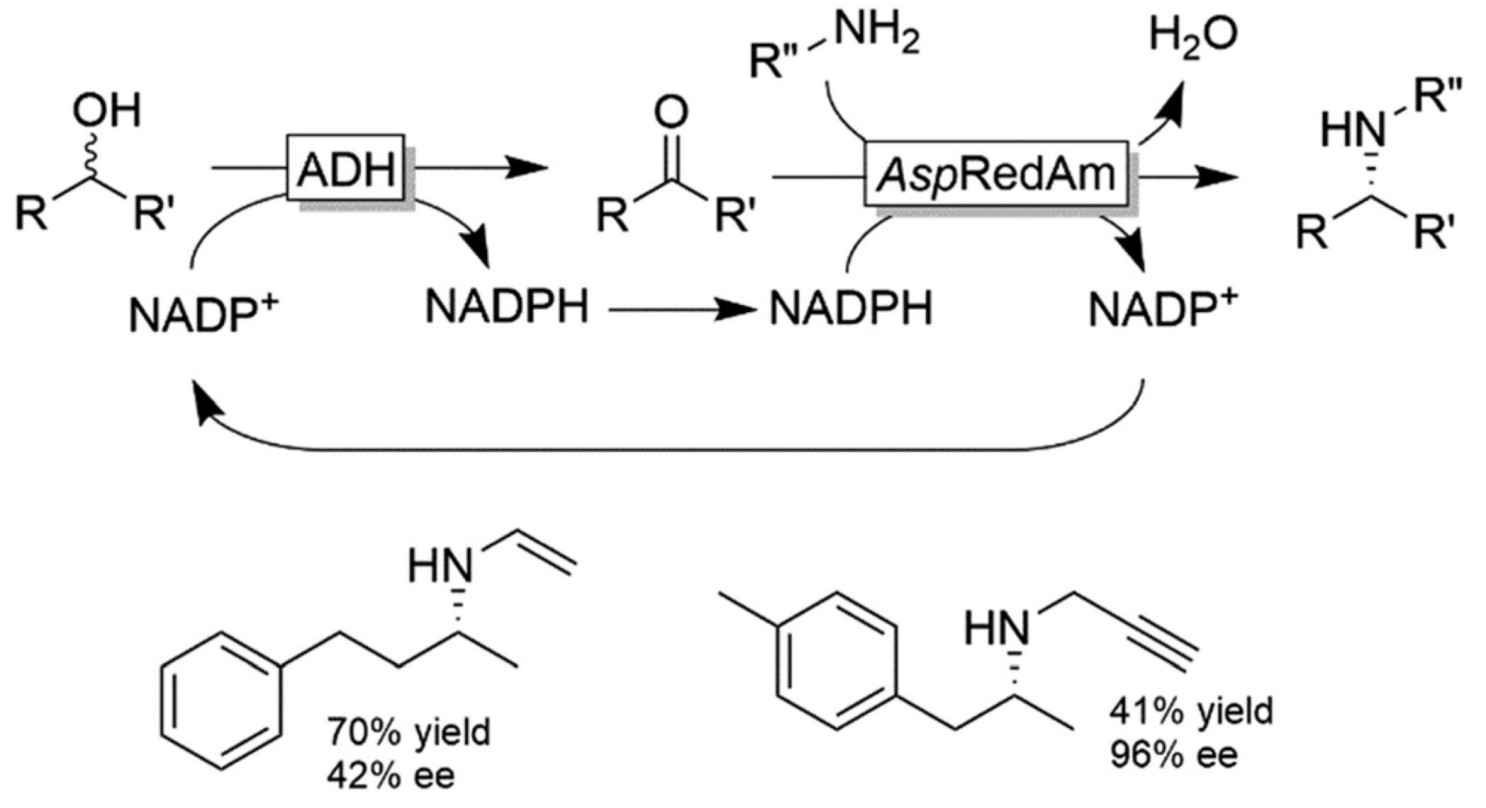
Deracemisation of secondary alcohols (using a non-stereoselective ADH) coupled to reductive amination with reductive aminases (*e.g*., from *Aspergillus*, *Asp*RedAm). ADHs used in this study were W110A TeSADH and the ADH from *Sphingobium yanoikuyae (Sy*ADH).

Recently, Turner and coworkers extended the applicability of the racemic alcohol bioamination cascade to the generation of secondary amines by replacing the AmDH with an im-ine reductase, from the “reductive aminase” sub-family (Scheme 26).^109^

**Scheme 27:**
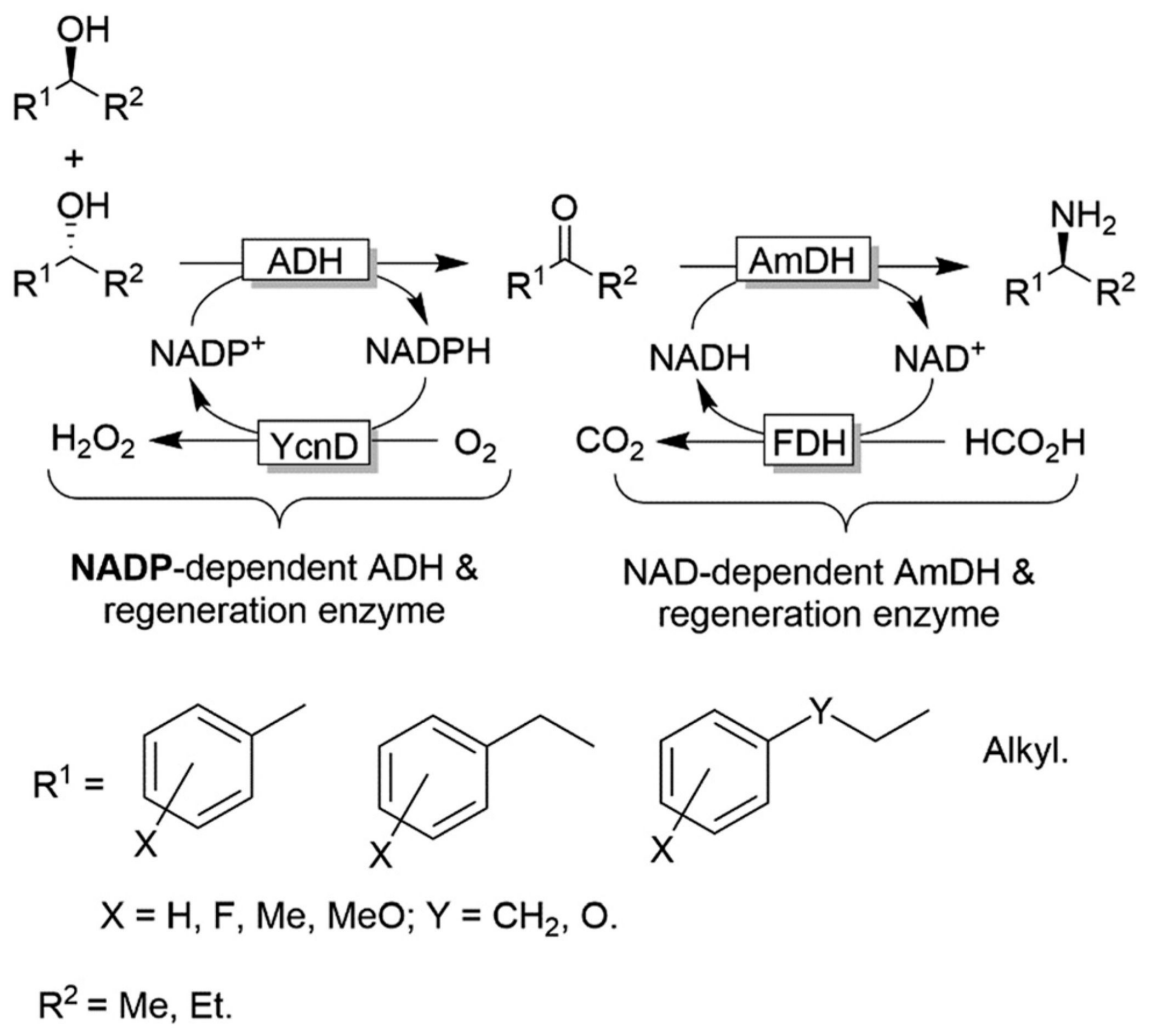
Orthogonal network for the deracemisation of secondary alcohols (using a non-enantioselective ADH) coupled to reductive amination with an amine dehydrogenase. All the enzymatic reactions are running simultaneously in one-pot.

Notably, in all studies mentioned above, the conversion obtained for the amination of racemic alcohols into the optically active amines never exceeded the threshold value of 95-96% although a high concentration of ammonium/ammonia buffer was applied *(ca.* 2 M). In contrast, primary alcohols could be aminated quantitatively. Mutti and coworkers explained these findings with a more unfavourable thermodynamic equilibrium for the amination of secondary alcohols compared to primary alcohols.^112^ Therefore they proposed an alternative orthogonal network in which a single NADP-dependent ADH (*i.e*., I86A W110A *Te*SADH variant, among the least selective mutants of *Te*SADH) oxidises the secondary alcohol to ketone, whereas a NAD-dependent *Am*DH performs the stereoselective reductive amination (Scheme 27). Orthogonal cofactor regeneration systems were applied in a similar way as for the deracemisation of secondary alcohols.^72^ Indeed, conversions above 99% was achieved with excellent enantiomeric excess (>99% R).

### Linear deracemisation of amines to optically active alcohols

**Scheme 28:**
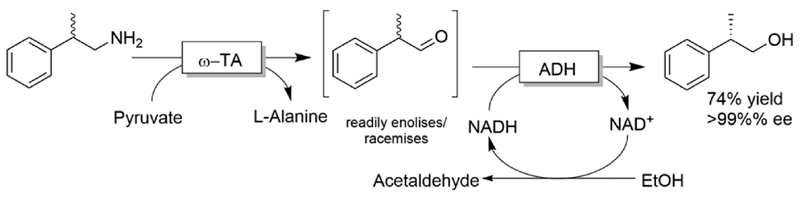
Deracemisation of (*R,S*)-2-phenylpropylamine to afford (*S*)-2-phenylpropanol using a non-stereoselective ω-TA coupled with an enantioselective ADH. The two steps were performed separately in a continuous flow system operating with a self-sustaining closed-loop.

The deracemisation of amines to yield optically active alcohols has attracted less interest until to date. Nevertheless, in some cases, racemic amines can be more accessible from natural resources than their structurally related alcohols. In a recent study, Contente and Paradisi performed the deracemisation of (*R,S*)-2-phenylpropylamine to afford (*S*)-2-phenylpropanol in 74% isolated yield and enantiomerically pure form (Scheme 28).^113^ The first step of the deracemisation is a non-stereoselective formal oxidative de-amination catalysed by a ω-TA, which consumes one equivalent of pyruvate. The racemic α-substituted aldehyde is then resolved in the second step since the applied alcohol dehydrogenase distinguishes between the two enantiomeric forms of the intermediate. As the unreacted aldehyde can also racemise spontaneously in solution, quantitative conversion into the final alcohol product can be achieved (*i.e*., DKR of the α-substituted aldehyde intermediate). Notably, the deracemisation sequence was carried out in a continuous flow system operating with an in-line recovery of products (including by-products) and a recirculation of the aqueous media that contains the cofactors. Thus, the ratio between cofactors (*i.e*., NAD, PLP) and substrates could be reduced from 1 :100 (typical for batch reactions) to 1:2000.

## Enantioconvergent deracemisation reactions

In enantioconvergent processes, the enantiomers of the starting material are transformed through inversion (undesired enantiomer) and retention (desired enantiomer) into an enantiomerically pure product (Scheme 2).^114^

**Scheme 29:**
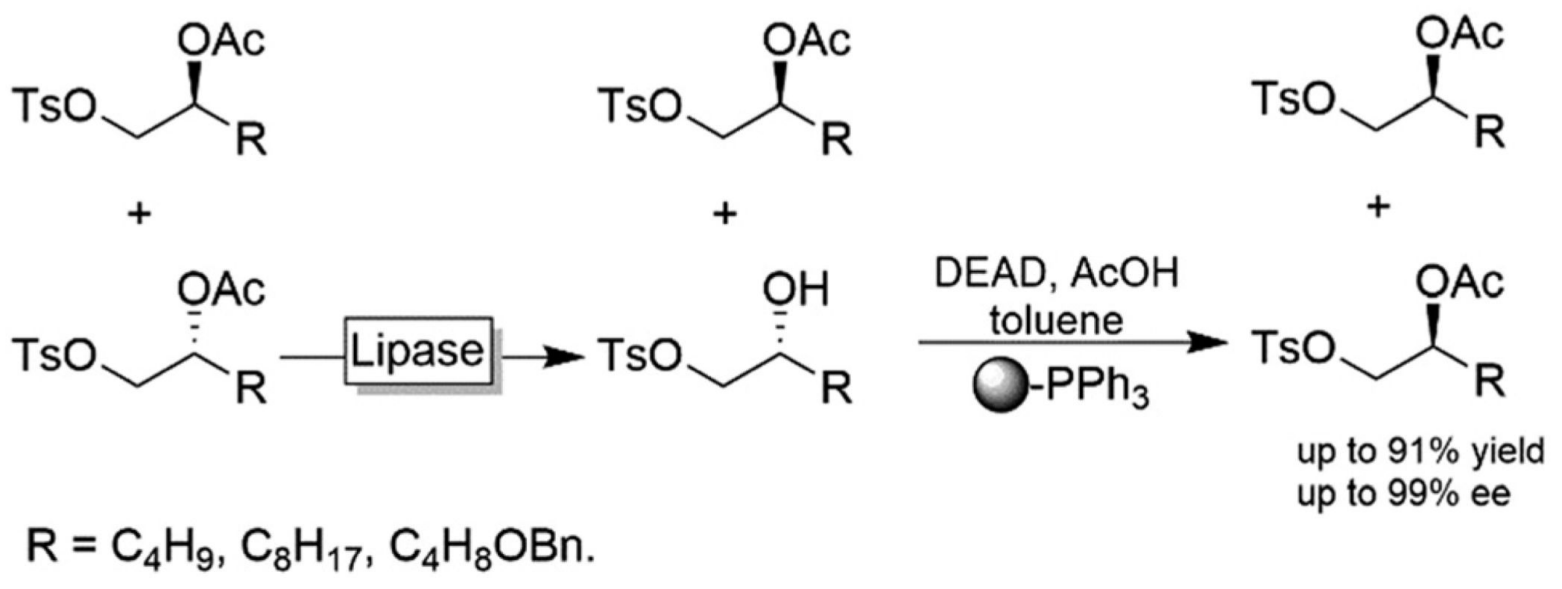
Chemoenzymatic enantioconvergent deracemisation of acetyl esters. Lipase PS from *Burkholderia cepacia* (EC 3.1.1.3), DEAD: diethyl azodicarboxylate.

An interesting chemoenzymatic enantioconvergent method was reported by Matsumoto and coworkers (Scheme 29).^115,116^ Racemic acetate esters were first submitted to a lipase-mediated KR followed by a chemical Mitsunobu reaction of the resulting alcohol enantiomer yielding essentially enantiomerically pure (*S*)-2-acetoxy tosylates. The tedious removal of non-reacted PPh_3_ from the Mitsunobu step was circumvented by using insoluble, polymer-supported PPh_3_.

**Scheme 30:**
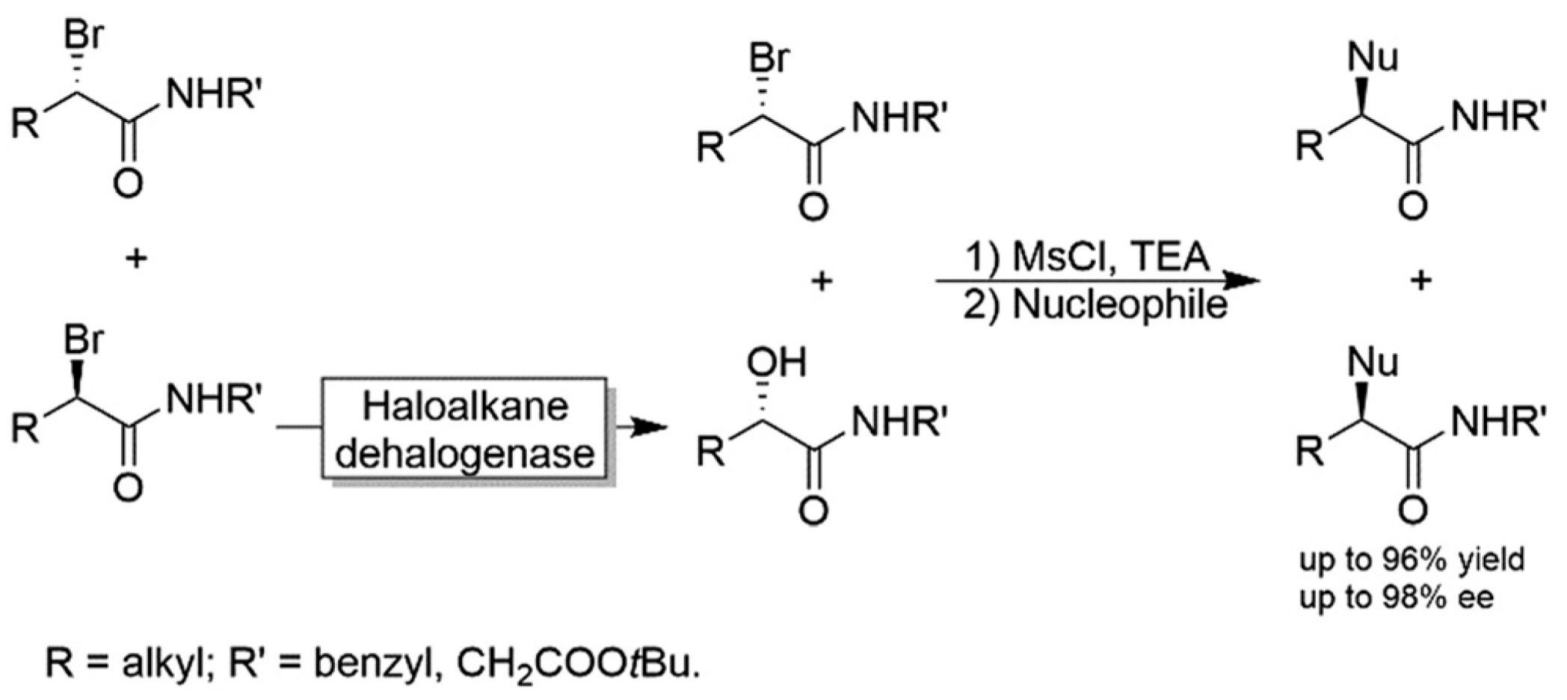
Chemo-enzymatic enantioconvergent synthesis of optically active α-substituted amides. Nucleophiles: Azide, benzylamine and phenol.

Another chemoenzymatic approach was suggested and implemented by Feringa and coworkers.^117^ α-Bromoamides were subjected to a KR catalysed by a haloalkane dehalogenase yielding the corresponding α-hydroxyamide through inversion of the configuration (*i.e*., the non-reacted starting material and the product were of the same configuration). After converting the alcohol into a leaving group by mesylation, both the unreacted bromide and the mesylated alcohol were subjected to S_N_2-type nucleophilic substitution by various nucleophiles including azide, benzylamine and phenol (Scheme 30).

**Scheme 31:**
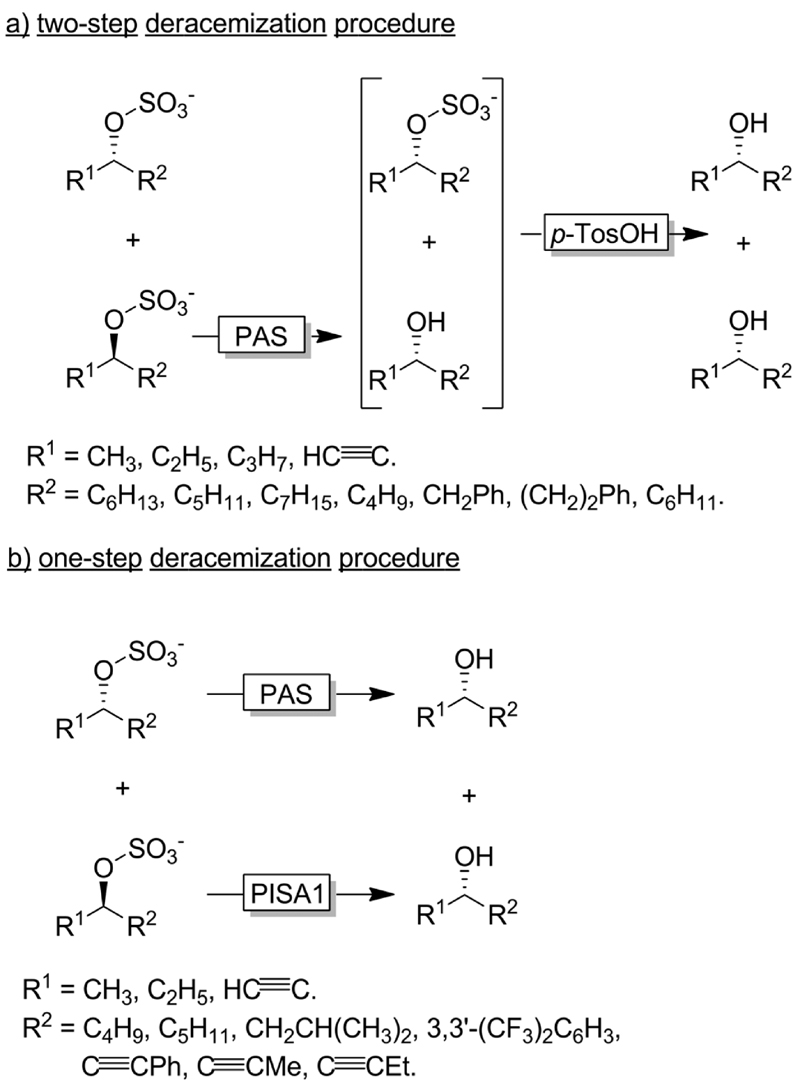
Deracemisation of sulfonate esters using an enantioconvergent chemoenzymatic two-step procedure (a) and a bienzymatic one-step procedure (b). PAS: Sulfatase from *Pseudomonas aeruginosa* (EC 3.1.6.1), PISA1: sulfatase from *Rhodopirellula baltica*.

Faber and coworkers have established alkyl sulfatases as enantioselective catalysts for the preparation of enantiomerically pure alcohols from racemic sulfate esters.^118–120^ In a first set of experiments the authors used an inverting sulfatase (*i.e*., catalysing the nucleophilic substitution at the C-atom) from *Pseudomonas aeruginosa* (PAS) thereby mediating a KR and leading to a 1 :1 mixture of the homochiral alcohol and sulfonate ester (Scheme 31a). Acidic hydrolysis lead to the deracemised alcohol.^119,121^ More elegantly, the Faber group later reported on the discovery of an enantio-complementary and retaining sulfatase (*i.e*., catalysing the nucleophilic substitution at the S-atom of the sulfonate group) from *Rhodopirellula baltica* (PISA1). A combination of both sulfatases enabled the one-step synthesis of various enantiopure alcohols from their sec-alkylsulfates (Scheme 31b).^118^

**Scheme 32:**
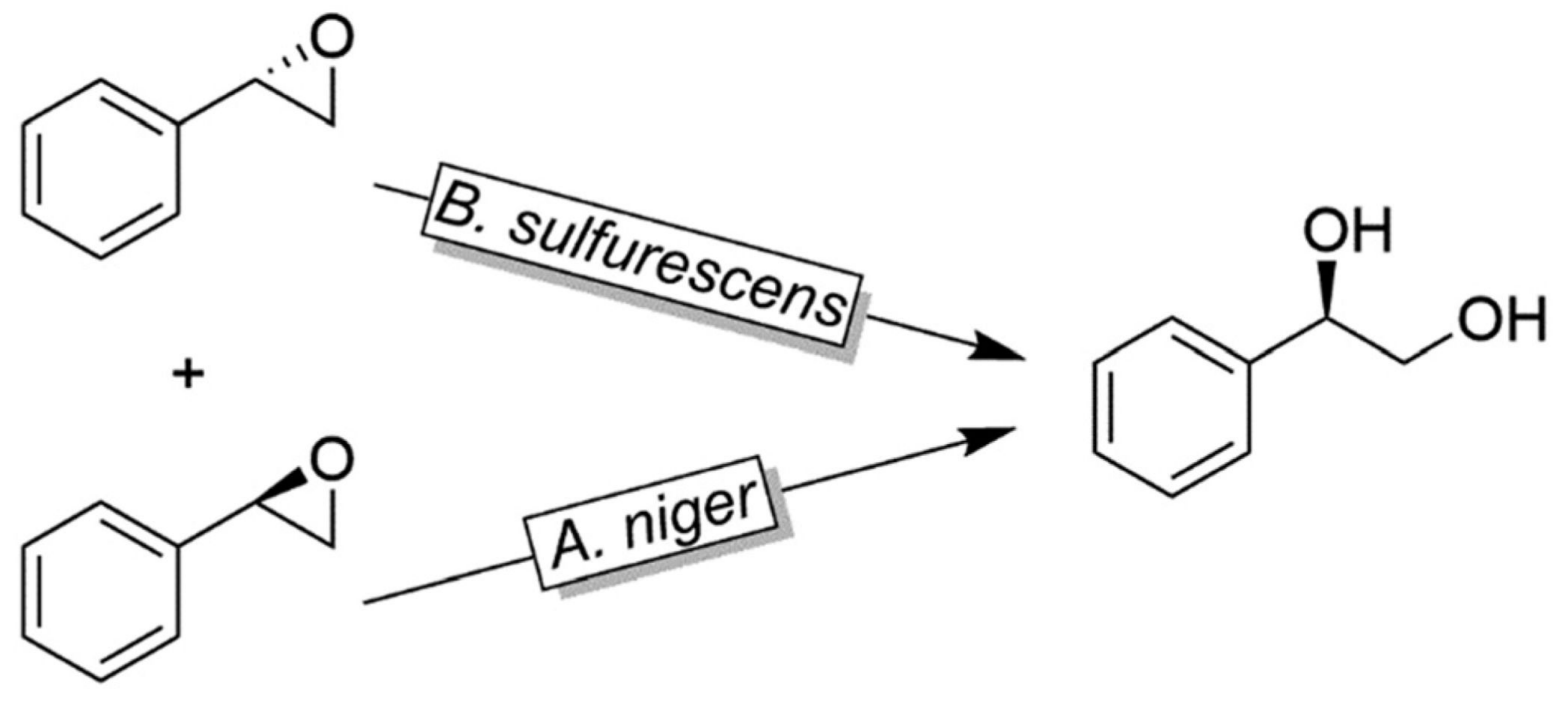
Deracemisation of styrene oxide using enantiocomplementary microbial strains (*Aspergillus niger* and *Beauveria sulfurescens*).

Like alkylsulfatases, epoxide hydrolases (EHs) can act on their substrates in Markovnikov- or *anti*-Markovnikov modes thereby leading to inversion or retention, respectively, of the absolute configuration. For example, as early as 1996, Furstoss and coworkers reported on two microbial strains (*Aspergillus niger and Beauveria sulfurescens*) exhibiting complementary EH activities resulting in the deracemisation of *e.g*. styrene oxide (Scheme 32).^122^ This enantioconvergence is due to a Markovnikov-attack catalysed by B. *sulfurescens* to (*S*)-styrene oxide leading to the (*R*)-diol combined with an *anti*-Markovnikov-attack by A. *niger* to the (*R*)-epoxide thereby retaining the absolute configuration.

**Scheme 33:**
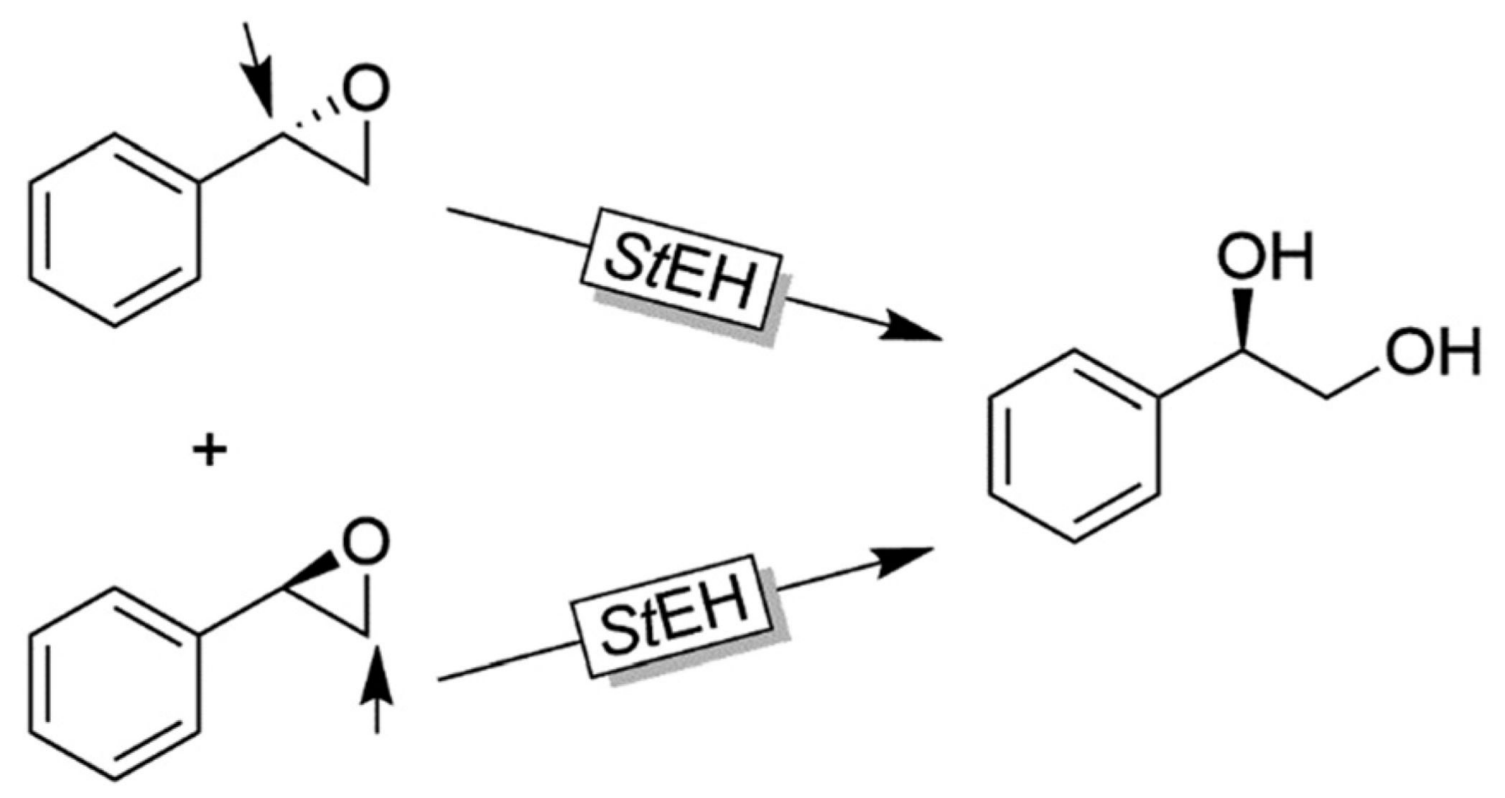
Deracemisation of styrene oxide using a single epoxide hydrolase. StEH: Epoxide hydrolase from *Solanum tuberosum* (EC 3.3.2.3).

Further examples with isolated enzymes have been reported ever since.^123,124^ An interesting alternative to the aforementioned bienzymatic EH-based deracemisation processes is to use EHs acting on both enantiomers of an oxirane albeit in Markovnikov and *anti*-Markovnikov selectivity. Next to the example by Furstoss and coworkers using the EH from *Solanum tuberosum* (*St*EH, Scheme 33),^125^ further EHs and their use in corresponding single enzymatic enantioconvergent reactions have been reported.^126–130^

## Conclusions

Deracemisation is a powerful approach for the synthesis of enantiopure alcohols and amines. This field has significantly advanced in the last few years, thus generating viable alternatives to the established kinetic resolution approaches.

The chemoenzymatic DKR of non-activated alcohols has reached maturity. More recently, bienzymatic DKR are moving into the focus. The promise is more compatible catalysts and simpler reaction schemes. A shortcoming for DKR is that the majority of the reported examples yield the (*R*)-configured product because they principally rely on a resolution step catalysed by well-known lipases that exhibit (*R*)-stereoselectivity. A larger portfolio of (*S*)-selective hydrolases will certainly boost the scope of DKRs. Fewer examples are known on DKR of amines because of the harsher conditions required for the racemisation of amines. Moreover, a bienzymatic DKR of amines is not reported yet. Other deracemisation strategies have the advantage of producing enantiopure alcohols or amines from their racemates without the need for an extra hydrolytic step (*e.g*., de-acylation).

Chemoenzymatic cyclic deracemisation of amines by combining a stereospecific oxidation and a non-stereoselective reduction is an appealing approach. However, the same approach is not well-known for deracemisation of alcohols. Deracemisation *via* stereoinversion is another promising strategy for obtaining enantiopure alcohols and amines for the advantage of avoiding futile cycles that are encountered in cyclic deracemisation. The challenge in this strategy is to design two stereocomplementary redox reactions that are compatible and orthogonal. When the latter condition is not possible, the deracemisation can still be conducted in one pot, but in two steps (*i.e*., linear deracemisation). Chemoenzymatic and bienzymatic sequential deracemisation *via* non-stereoselective oxidation along with a stereoselective reduction has also been reported. More efforts should be devoted in designing orthogonal redox reactions that enable a one-pot, one-step deracemisation approaches.

Hydrogen borrowing approaches to produce enantiopure amines from their racemic alcohols have also been reported. Although resolved in few cases, the design of an orthogonal network remains the critical point in this strategy.

Most of the reported deracemisation strategies for alcohols rely on formation of carbonyl-containing intermediates, and thus makes the deracemisation of tertiary alcohols a challenging problem. Enzymatic or chemical racemisation methods for tertiary alcohols, which are also compatible with a simultaneous enzymatic resolution step, should be developed in order to enable the development of efficient enzymatic or chemo-enzymatic deracemisation approach for tertiary alcohols. Overall, another common difficulty encountered in enzyme-based deracemisation approaches of alcohols and amines is the relatively narrow substrate scopes of enzymes when compared with organometallic- or organo-catalysis. Recent advances in protein engineering including directed evolution and structure-guided enzyme design will allow for filling these gaps by creating new enzyme variants possessing the desired and wider substrate scope, and controlled enantioselectivity. The future will tell if the promise of more efficient deracemisation methods will be kept on industrial scale.

## References

[1] Goldberg K, Schroer K, Lutz S, Liese A (2007). Appl Microbiol Biotechnol.

[2] Kroutil W, Mang H, Edegger K, Faber K (2004). Curr Opin Chem Biol.

[3] Gomm A, O’Reilly E (2018). Curr Opin Chem Biol.

[4] Grogan G (2018). Curr Opin Chem Biol.

[5] Ghislieri D, Turner NJ (2014). Top Catal.

[6] Patil MD, Grogan G, Bommarius A, Yun H (2018). ACS Catal.

[7] Kohls H, Steffen-Munsberg F, Höhne M (2014). Curr Opin Chem Biol.

[8] Catani M, Ismail OH, Gasparrini F, Antonelli M, Pasti L, Marchetti N, Felletti S, Cavazzini A (2017). Analyst.

[9] Kellogg M, Nieuwenhuijzen JW, Pouwer K, Vries TR, Broxterman QB, Grimbergen RFP, Kaptein B, Crois RML, Wever Ed, Zwaagstra K, Laan Acvd (2003). Synthesis.

[10] Nugent TC (2010). Chiral amine synthesis: Methods, Developments and Applications.

[11] Wang C, Xiao J, Li W, Zhang X (2014). Stereoselective Formation of Amines.

[12] Sharma M, Mangas-Sanchez J, Turner NJ, Grogan G (2017). Adv Synth Catal.

[13] Chen Q-A, Ye Z-S, Duan Y, Zhou Y-G (2013). Chem Soc Rev.

[14] Cho BT (2009). Chem Soc Rev.

[15] Slabu I, Galman JL, Lloyd RC, Turner NJ (2017). ACS Catal.

[16] Torrelo G, Hanefeld U, Hollmann F (2015). Catal Lett.

[17] Nestl BM, Hammer SC, Nebel BA, Hauer B (2014). Angew Chem, Int Ed.

[18] Bornscheuer U, Kazlauskas R (2006). Hydrolases in Organic Synthesis.

[19] Hilterhaus L, Liese A, Ulber R, Sell D (2007). White Biotechnology.

[20] Liese A, Seelbach K, Wandrey C (2006). Industrial Biotransformations.

[21] Patil MD, Grogan G, Bommarius A, Yun H (2018). Catalysts.

[22] Huisman GW, Collier SJ (2013). Curr Opin Chem Biol.

[23] Huisman GW, Liang J, Krebber A (2010). Curr Opin Chem Biol.

[24] Bornscheuer UT, Huisman GW, Kazlauskas RJ, Lutz S, Moore JC, Robins K (2012). Nature.

[25] Kelly SA, Pohle S, Wharry S, Mix S, Allen CCR, Moody TS, Gilmore BF (2018). Chem Rev.

[26] Schrittwieser JH, Velikogne S, Kroutil W (2015). Adv Synth Catal.

[27] Fuchs M, Farnberger JE, Kroutil W (2015). Eur J Org Chem.

[28] Guo F, Berglund P (2017). Green Chem.

[29] Alba D-R, Ivan L, Vicente G (2015). Curr Green Chem.

[30] Rachwalski M, Vermue N, Rutjes FPJT (2013). Chem Soc Rev.

[31] Steinreiber J, Faber K, Griengl H (2008). Chem – Eur J.

[32] Diaz-Rodriguez A, Lavandera I, Gotor V (2015). Curr Green Chem.

[33] Applegate GA, Berkowitz DB (2015). Adv Synth Catal.

[34] Cheng-jun J, Guilin C (2013). Curr Org Chem.

[35] Turner NJ (2008). Asymmetric Organic Synthesis with Enzymes.

[36] Tessaro D, Molla G, Pollegioni L, Servi S (2009). Modern Biocatalysis.

[37] Verho O, Bäckvall JE (2015). J Am Chem Soc.

[38] Allen JV, Williams JMJ (1996). Tetrahedron Lett.

[39] Persson BA, Larsson ALE, Le Ray M, Bäckvall JE (1999). J Am Chem Soc.

[40] Larsson ALE, Persson BA, Bäckvall JE (1997). Angew Chem, Int Ed Engl.

[41] Pellissier H (2016). Tetrahedron.

[42] Langvik O, Saloranta T, Murzin DY, Leino R (2015). ChemCatChem.

[43] Kim Y, Park J, Kim M-J (2011). ChemCatChem.

[44] Conley BL, Pennington-Boggio MK, Boz E, Williams TJ (2010). Chem Rev.

[45] Choi JH, Choi YK, Kim YH, Park ES, Kim EJ, Kim M-J, Park J (2004). J Org Chem.

[46] Choi JH, Kim YH, Nam SH, Shin ST, Kim M-J, Park J (2002). Angew Chem, Int Ed.

[47] Haak RM, Berthiol F, Jerphagnon T, Gayet AJA, Tarabiono C, Postema CP, Ritleng V, Pfeffer M, Janssen DB, Minnaard AJ, Feringa BL (2008). J Am Chem Soc.

[48] Strauss UT, Faber K (1999). Tetrahedron: Asymmetry.

[49] Gruber CC, Nestl BM, Gross J, Hildebrandt P, Bornscheuer UT, Faber K, Kroutil W (2007). Chem – Eur J.

[50] Musa MM, Patel JM, Nealon CM, Kim CS, Phillips RS, Karume I (2015). J Mol Catal B: Enzym.

[51] Musa MM, Ziegelmann-Fjeld KI, Vieille C, Phillips RS (2008). Org Biomol Chem.

[52] Karume I, Musa MM, Bsharat O, Takahashi M, Hamdan SM, El Ali B (2016). RSC Adv.

[53] Popłoński J, Reiter T, Kroutil W (2018). ChemCatChem.

[54] Mutti FG, Orthaber A, Schrittwieser JH, Vries JGd, Pietschnig R, Kroutil W (2010). Chem Commun.

[55] Magallanes-Noguera C, Ferrari MM, Kurina-Sanz M, Orden AA (2012). J Biotechnol.

[56] Tanaka T, Iwai N, Matsuda T, Kitazume T (2009). J Mol Catal B: Enzym.

[57] Escalettes F, Turner NJ (2008). ChemBioChem.

[58] Turner NJ (2010). Curr Opin Chem Biol.

[59] Alexeeva M, Enright A, Dawson MJ, Mahmoudian M, Turner NJ (2002). Angew Chem, Int Ed.

[60] Herter S, Medina F, Wagschal S, Benhaïm C, Leipold F, Turner NJ (2018). Bioorg Med Chem.

[61] Ghislieri D, Green AP, Pontini M, Willies SC, Rowles I, Frank A, Grogan G, Turner NJ (2013). J Am Chem Soc.

[62] Rowles I, Malone KJ, Etchells LL, Willies SC, Turner NJ (2012). ChemCatChem.

[63] Dunsmore CJ, Carr R, Fleming T, Turner NJ (2006). J Am Chem Soc.

[64] Carr R, Alexeeva M, Dawson MJ, Gotor-Fernández V, Humphrey CE, Turner NJ (2005). ChemBioChem.

[65] Heath RS, Pontini M, Bechi B, Turner NJ (2014). ChemCatChem.

[66] Yasukawa K, Nakano S, Asano Y (2014). Angew Chem, Int Ed.

[67] Foulkes JM, Malone KJ, Coker VS, Turner NJ, Lloyd JR (2011). ACS Catal.

[68] Schrittwieser JH, Groenendaal B, Resch V, Ghislieri D, Wallner S, Fischereder EM, Fuchs E, Grischek B, Sattler JH, Macheroux P, Turner NJ (2014). Angew Chem, Int Ed.

[69] Jeon H, Yoon S, Ahsan MM, Sung S, Kim G-H, Sundaramoorthy U, Rhee S-K, Yun H (2017). Catalysts.

[70] Aleku GA, Mangas-Sanchez J, Citoler J, France SP, Montgomery SL, Heath RS, Thompson MP, Turner NJ (2018). ChemCatChem.

[71] Mugford PF, Wagner UG, Jiang Y, Faber K, Kazlauskas RJ (2008). Angew Chem, Int Ed.

[72] Voss CV, Gruber CC, Faber K, Knaus T, Macheroux P, Kroutil W (2008). J Am Chem Soc.

[73] Paul CE, Lavandera I, Gotor-Fernández V, Kroutil W, Gotor V (2013). ChemCatChem.

[74] Xue Y-P, Zheng Y-G, Zhang Y-Q, Sun J-L, Liu Z-Q, Shen Y-C (2013). Chem Commun.

[75] Voss CV, Gruber CC, Kroutil W (2008). Angew Chem, Int Ed.

[76] Li B, Nie Y, Mu XQ, Xu Y (2016). J Mol Catal B: Enzym.

[77] Fragnelli MC, Hoyos P, Romano D, Gandolfi R, Alcantara AR, Molinari F (2012). Tetrahedron.

[78] Amrutkar SM, Banoth L, Banerjee UC (2013). Tetrahedron Lett.

[79] Venkataraman S, Chadha A (2015). J Ind Microbiol Biotechnol.

[80] Sivakumari T, Chadha A (2015). RSC Adv.

[81] Saravanan T, Selvakumar R, Doble M, Chadha A (2012). Tetrahedron: Asymmetry.

[82] Adam W, Lazarus M, Saha-Möller CR, Schreier P (1998). Tetrahedron: Asymmetry.

[83] Xue Y-P, Zeng H, Jin X-L, Liu Z-Q, Zheng Y-G (2016). Microb Cell Fact.

[84] Mendez-Sanchez D, Mangas-Sanchez J, Lavandera I, Gotor V, Gotor-Fernandez V (2015). ChemCatChem.

[85] Diaz-Rodriguez A, Rios-Lombardia N, Sattler JH, Lavandera I, Gotor-Fernandez V, Kroutil W, Gotor V (2015). Catal Sci Technol.

[86] Kedziora K, Diaz-Rodriguez A, Lavandera I, Gotor-Fernandez V, Gotor V (2014). Green Chem.

[87] Liardo E, Ríos-Lombardía N, Morís F, González-Sabín J, Rebolledo F (2018). Eur J Org Chem.

[88] Karume I, Takahashi M, Hamdan SM, Musa MM (2016). ChemCatChem.

[89] Musa MM, Karume I, Takahashi M, Hamdan SM, Ullah N (2018). ChemistrySelect.

[90] Koszelewski D, Pressnitz D, Clay D, Kroutil W (2009). Org Lett.

[91] Koszelewski D, Muller N, Schrittwieser JH, Faber K, Kroutil W (2010). J Mol Catal B: Enzym.

[92] Shin G, Mathew S, Shon M, Kim BG, Yun H (2013). Chem Commun.

[93] Han S-W, Jang Y, Shin J-S (2019). ACS Catal.

[94] O’Reilly E, Iglesias C, Turner NJ (2014). ChemCatChem.

[95] Seo Y-M, Mathew S, Bea H-S, Khang Y-H, Lee S-H, Kim B-G, Yun H (2012). Org Biomol Chem.

[96] Heath RS, Pontini M, Hussain S, Turner NJ (2016). ChemCatChem.

[97] Köhler V, Wilson YM, Durrenberger M, Ghislieri D, Churakova E, Quinto T, Knorr L, Haussinger D, Hollmann F, Turner NJ, Ward TR (2013). Nat Chem.

[98] Khorsand F, Murphy CD, Whitehead AJ, Engel PC (2017). Green Chem.

[99] Tauber K, Fuchs M, Sattler JH, Pitzer J, Pressnitz D, Koszelewski D, Faber K, Pfeffer J, Haas T, Kroutil W (2013). Chem – Eur J.

[100] Sattler JH, Fuchs M, Tauber K, Mutti FG, Faber K, Pfeffer J, Haas T, Kroutil W (2012). Angew Chem, Int Ed.

[101] Palacio CM, Crismaru CG, Bartsch S, Navickas V, Ditrich K, Breuer M, Abu R, Woodley JM, Baldenius K, Wu B, Janssen DB (2016). Biotechnol Bioeng.

[102] Lerchner A, Achatz S, Rausch C, Haas T, Skerra A (2013). ChemCatChem.

[103] Pickl M, Fuchs M, Glueck SM, Faber K (2015). ChemCatChem.

[104] Fuchs M, Tauber K, Sattler J, Lechner H, Pfeffer J, Kroutil W, Faber K (2012). RSC Adv.

[105] Martinez-Montero L, Gotor V, Gotor-Fernandez V, Lavandera I (2017). Green Chem.

[106] Mutti FG, Knaus T, Scrutton NS, Breuer M, Turner NJ (2015). Science.

[107] Chen FF, Liu YY, Zheng GW, Xu JH (2015). ChemCatChem.

[108] Böhmer W, Knaus T, Mutti FG (2018). ChemCatChem.

[109] Montgomery SL, Mangas-Sanchez J, Thompson MP, Aleku GA, Dominguez B, Turner NJ (2017). Angew Chem, Int Ed.

[110] Thompson MP, Turner NJ (2017). ChemCatChem.

[111] Resch V, Fabian WMF, Kroutil W (2010). Adv Synth Catal.

[112] Knaus T, Cariati L, Masman MF, Mutti FG (2017). Org Biomol Chem.

[113] Contente ML, Paradisi F (2018). Nat Catal.

[114] Schober M, Faber K (2013). Trends Biotechnol.

[115] Matsumoto K, Usuda K, Okabe H, Hashimoto M, Shimada Y (2013). Tetrahedron: Asymmetry.

[116] Shimada Y, Usuda K, Okabe H, Suzuki T, Matsumoto K (2009). Tetrahedron: Asymmetry.

[117] Szymanski W, Westerbeek A, Janssen DB, Feringa BL (2011). Angew Chem, Int Ed.

[118] Schober M, Toesch M, Knaus T, Strohmeier GA, van Loo B, Fuchs M, Hollfelder F, Macheroux P, Faber K (2013). Angew Chem, Int Ed.

[119] Schober M, Gadler P, Knaus T, Kayer H, Birner-Grunberger R, Gully C, Macheroux P, Wagner U, Fabert K (2011). Org Lett.

[120] Simeo Y, Faber K (2006). Tetrahedron: Asymmetry.

[121] Fuchs M, Schober M, Pfeffer J, Kroutil W, Birner-Gruenberger R, Faber K (2011). Adv Synth Catal.

[122] Pedragosa Moreau S, Archelas A, Furstoss R (1996). Tetrahedron.

[123] Hwang S, Choi CY, Lee EY (2008). Biotechnol Lett.

[124] Hwang S, Choi CY, Lee EY (2008). Biotechnol Bioprocess Eng.

[125] Monterde MI, Lombard M, Archelas A, Cronin A, Arand M, Furstoss R (2004). Tetrahedron: Asymmetry.

[126] Steinreiber A, Mayer SF, Saf R, Faber K (2001). Tetrahedron: Asymmetry.

[127] Lin SJ, Horsman GP, Chen YH, Li WL, Shen B (2009). J Am Chem Soc.

[128] Zhu Q-Q, He W-H, Kong X-D, Fan L-Q, Zhao J, Li S-X, Xu J-H (2014). Appl Microbiol Biotechnol.

[129] Wu Y-W, Kong X-D, Zhu Q-Q, Fan L-Q, Xu J-H (2015). Catal Commun.

[130] Li F-L, Kong X-D, Chen Q, Zheng Y-C, Xu Q, Chen F-F, Fan L-Q, Lin G-Q, Zhou J, Yu H-L, Xu J-H (2018). ACS Catal.

